# Circulating immunoregulatory B cell and autoreactive antibody profiles predict lack of toxicity to anti-PD-1 checkpoint inhibitor treatment in advanced melanoma

**DOI:** 10.1136/jitc-2025-011682

**Published:** 2025-05-31

**Authors:** Zena N Willsmore, Lucy Booth, Akshay Patel, Ashley Di Meo, Ioannis Prassas, Jitesh Chauhan, Yin Wu, Amanda Fitzpartick, Katie Stoker, Matthaios Kapiris, Dhruva Biswas, Esperanza Perucha, Sean Whittaker, Sophia Tsoka, Eleftherios P Diamandis, Gary W Middleton, Thomas J Tull, Sophie Papa, Katie E Lacy, Sophia N Karagiannis

**Affiliations:** 1 St John's Institute of Dermatology, School of Basic and Medical Biosciences and KHP Centre for Translational Medicine, Guy’s Hospital, King’s College London, London, UK; 2 Institute of Immunology and Immunotherapy (III), College of Medicine and Health, University of Birmingham, Birmingham, UK; 3 Department of Laboratory Medicine and Pathobiology, University of Toronto, Toronto, Ontario, Canada; 4 Division of Clinical Biochemistry, Laboratory Medicine Program, Toronto General Hospital, University Health Network, Toronto, Ontario, Canada; 5 Laboratory Medicine Program, University Health Network, Toronto, Ontario, Canada; 6 Department of Medical Oncology, Guy's and St Thomas’ Hospitals NHS Trust, London, UK; 7 Centre for Inflammation Biology and Cancer Immunology, School of Immunology and Microbial Sciences, King’s College London, London, UK; 8 Breast Cancer Now Research Unit, School of Cancer and Pharmaceutical Sciences, Innovation Hub, Guy’s Cancer Centre, King’s College London, London, UK; 9 Department of Informatics, Faculty of Natural, Mathematical and Engineering Sciences, King’s College London, Bush House, Strand Campus, King’s College London, London, UK; 10 Comprehensive Cancer Centre, School of Cancer and Pharmaceutical Sciences, Innovation Hub, Guy’s Cancer Centre, King’s College London, London, UK; 11 Cardiovascular Data Science (CarDS) Lab, Research Faculty, Yale School of Medicine, New Haven, Connecticut, USA; 12 School of Cardiovascular and Metabolic Medicine & Sciences, James Black Centre, King’s College London, London, UK; 13 Centre for Rheumatic Diseases, King’s College London, London, UK; 14 Lunenfeld-Tanenbaum Research Institute, Mount Sinai Hospital, Toronto, Ontario, Canada; 15 Department of Pathology and Laboratory Medicine, Mount Sinai Hospital, Toronto, Ontario, Canada; 16 St John’s Institute of Dermatology, Guy's and St Thomas’ NHS Foundation Trust, London, UK

**Keywords:** B cell, Immune Checkpoint Inhibitor, Autoimmune, Humoral, Skin Cancer

## Abstract

**Background:**

The majority of patients with melanoma develop immune-related adverse events (irAEs), and over half do not respond to anti-PD-1 (Programmed cell death protein 1) checkpoint inhibitor (CPI) immunotherapy. Accurate predictive biomarkers for both response to therapy and development of irAEs are currently lacking in clinical practice. Here, we conduct deep immunophenotyping of circulating regulatory and class-switched B cell and antibody immune states in patients with advanced stage III/IV melanoma prior to and longitudinally during CPI.

**Methods:**

Mass cytometry, serum antibody isotyping and immuno-mass spectrometry proteome-wide screening evaluations to identify autoreactive antibodies were undertaken to profile circulating humoral immunity features in patients and healthy subjects and interrogate pretreatment B cell and antibody signatures that predict toxicity and response to anti-PD-1 therapy. In paired blood samples pretreatment and post-treatment, these humoral immune response profiles were monitored and correlated with the onset of toxicity.

**Results:**

We found increased circulating IL-10+ (Interleukin-10+) plasmablasts and double-negative (DN) B cell frequencies, higher PD-L1 (programmed death ligand 1), TGFβ (Transforming Growth Factorβ) and CD95 expression by B cells, alongside higher IgG4 and IgE serum levels in patients with stage III/IV melanoma. This suggests enhanced B regulatory and Th2 (Thelper2)-driven responses in advanced disease. Increased baseline frequency of DN2 B cells, plasmablasts, and serum IgE, IgA and antibody autoreactivity were observed in patients who did not develop irAE. During treatment, higher IL-10+class-switched memory B cell, plasmablast and IgG1, IgG3 and IgE, alongside reduced IgG2, IgG4, IgA and IgM levels, were observed. A reduction in autoantibodies targeting tubulins was observed during treatment. Increased frequency of class-switched memory B cells predicted improved survival, while reduced transitional and PD-L1+TGFβ+ naive B cell frequencies and higher IgG4 and IgE levels predicted lower survival, on anti-PD-1 therapy.

**Conclusions:**

Distinct B cell and antibody reactivities in patients with advanced melanoma share features with extrafollicular B cell responses in autoimmune diseases, may be protective from irAE and help predict outcomes to anti-PD-1.

**WHAT IS ALREADY KNOWN ON THIS TOPIC**

- The development of immune-related adverse events (irAEs) is a major challenge in the clinical management of patients receiving anti-PD-1 (Programmed cell death protein 1) checkpoint inhibitors (CPI).

**WHAT THIS STUDY ADDS**

- Our study reveals a skewed naïve and alternatively-activated circulating B cell and antibody compartment in melanoma and identifies regulatory and autoreactive features in patients that protect from CPI-induced irAEs. Class-switched humoral immune phenotypes associate with more favourable treatment response in advanced melanoma.

**HOW THIS STUDY MIGHT AFFECT RESEARCH, PRACTICE OR POLICY**

- Circulating humoral profiles in advanced melanoma may help predict and monitor toxicity and treatment response, harbouring potential to facilitate stratification a priori, and identify patients for close monitoring and early intervention of irAEs.

## Introduction

 Checkpoint inhibitors (CPI) targeting T cell functions, particularly those directed to the PD-1 (Programmed cell death protein 1) axis, constitute a paradigm shift for patient outcomes in advanced melanoma. Despite considerable success, a key consideration is recognizing which patients will develop immune-related adverse events (irAEs), which can affect any organ and vary in severity, but commonly target the skin, the gastrointestinal and the endocrine systems.^1^ Although many toxicities are manageable,^2^ some associate with significant long-term morbidity and necessitate treatment interruption or discontinuation in~20% of patients.^3^ Currently, there are no predictive biomarkers for irAEs in clinical use. As only half of patients respond to therapy,^4^ this represents an unmet need to predict and monitor therapy response.

Although melanoma is highly immunogenic, immune evasion mechanisms can modulate both T and B cell functions.^5^ Exposure to the tumor microenvironment (TME) may alter B cell maturation and skew antibody class-switching to less effective isotypes that favor tumor progression^6^ and could influence therapy response. Induction of regulatory B cell (Breg) phenotypes, characterized by IL-10 (Interleukin-10), TGFβ (Transforming Growth Factorβ), PD-1 and PD-L1 (Programmed death ligand 1) expression, may be important components of immune responses in models of cancer and in patients.^7^ Enriched differentiated B cell phenotypes such as memory, plasmablasts and plasma cells have been reported within melanoma lesions.^6 8^ Plasmablasts are short-lived cells and participate in rapid antigen responses. Their functional plasticity may allow transient switching to a “regulatory” pro-tumor phenotype owing to expression of cytokines such as TGFβ and IL-10, signaling modulatory states that could potentially influence clinical outcomes including toxicity and therapy response. Melanoma may promote regulatory and alternatively activated B cell responses, including in patient circulation. Enriched circulating and tumor-infiltrating TGFβ+ B cells in advanced melanoma favor FoxP3+ Treg (Forkhead box P3+ regulatory T cell) expansion in a TGFβ-dependent manner,^7^ and IgG4+CD49b+CD73+ Bregs (regulatory B cells) produce pro-angiogenic and inflammatory mediators (eg, vascular endothelial growth factor).^9^ Bregs may thus engender multiple inhibitory functions supporting melanoma progression and differential therapy outcomes in advanced disease.

B cell-produced antibodies mediate antibody-dependent cellular cytotoxicity, antibody-dependent cellular phagocytosis and complement-dependent cytotoxicity. These depend on antibody isotype, specificity and affinity for their target antigen. Increased immunoglobulin expression in the TME has been linked with improved survival in melanoma and other solid tumors. However, tumor-associated immune suppression and Th2 (Thelper2)-biased conditions may skew class-switching to isotypes such as IgG4 likely to curtail immune stimulation.^810^,^12^ Furthermore, autoreactivity is recognized in melanoma clinically, in the form of vitiligo,^13^ linked with more favorable prognosis.^14^ Circulating antibodies in advanced melanoma display differential reactivity profiles, including to cancer antigens,^15^ cancer testis antigens^16^ and numerous self-antigens, demonstrating parallel features with those reported in autoimmune disease.^17^

While the humoral compartment could mediate several mechanisms to promote or halt melanoma growth and progression, in-depth characterizations of circulating B cells in relation to CPI toxicity and response remain limited. We hypothesize that Breg phenotypes and antibody isotypes associate with less potent immune responses, and certain features of these may moderate the toxic effects of immunotherapies. Contrastingly, augmented class-switched B cells and antibodies may signal an active and anticipatory response to be harnessed by immunotherapy. Here, we undertake deep immunophenotyping of circulating B cell and antibody immune states in patients with advanced stage III/IV melanoma in the context of anti-PD-1 CPI therapy, by mass cytometry (cytometry by time of flight, CyTOF), antibody isotyping and proteome-wide autoantigen discovery using immuno-mass spectrometry (IMS). We characterize circulating humoral immunity features in patients and healthy subjects and interrogate pretreatment signatures that predict toxicity and response to anti-PD-1 (CPI) therapy. In paired samples pretreatment and post-treatment, we monitor the humoral immune response during CPI and correlate this with toxicity and outcomes.

## Materials and methods

### Sample collection

Whole blood samples were collected in yellow-capped serum separating collection tubes and allowed to clot for a minimum of 30 min. Peripheral blood mononuclear cell isolation and cryopreservation were performed as before^17^,^19^ (online supplemental materials and methods).

### Patient characteristics

52 patients with stage III/IV melanoma were recruited who were planned to receive anti-PD-1 monotherapy in adjuvant and active disease settings (online supplemental table 1). Baseline peripheral blood sampling was performed pretreatment (median 14 days prior to first dose, online supplemental table 1). On-treatment sampling was performed according to treatment protocol (pembrolizumab: week 6 and week 12; nivolumab: week 8 and week 12) (online supplemental figure 1). irAE grading was recorded as per ESMO (European Society for Medical Oncology) clinical guidelines applied by consultant oncologists involved in patient care, and “high-grade” toxicity was defined as grade 3 or higher irAE (ESMO clinical guidelines)^20^ (online supplemental table 2). To define toxicity outcomes, patients were designated as “no toxicity”, “high-grade toxicity” or “all toxicity” (all patients with any grade toxicity, encompassing grade 1–3 irAE). Only “high-grade toxicity” patients were included in the toxicity cohort to allow clearer identification of B cell subsets most likely to predispose to severe toxicity that warranted treatment discontinuation (online supplemental table 2). Treatment was withheld due to irAEs in all “high-grade” toxicity patients (online supplemental figure 1). Statistical analyses are described in online supplemental materials and methods. We used the STROBE (Strengthening the Reporting of Observational Studies in Epidemiology) case–control checklist.

### Mass cytometry and cluster analysis using the FlowSOM clustering algorithm

CyTOF was conducted for B cell subset analysis (online supplemental figure 1) to enable in-depth and high-dimensional immunophenotyping (gating strategy: online supplemental figure 2) (online supplemental materials and methods). A previously described marker panel was adapted (online supplemental table 3).^17 19^

### Multi-plex immunoassay for serum antibody isotyping

A high-throughput antibody isotyping magnetic bead-based assay of seven antibody isotypes (IgG1, IgG2, IgG3, IgG4, IgE, IgM, IgA) was performed (Thermo Fisher).^21^ Samples were collected, stored at −80°C and thawed on ice prior to assay. 10 µl serum per patient, per assay, was diluted at concentrations: 1:60,000 for IgG1 and IgG3; 1:20,000 for IgG2, IgG3, IgG4, IgE, IgM and IgA.^21^ Data was acquired using the FLEXMAP3D (Luminex) analyzer. Output files were checked for accuracy of standard curves and data extracted for analyses.

### Immuno-mass spectrometry coupled to liquid chromatography to identify serum autoreactive antibodies

Serum samples were evaluated by liquid chromatography coupled to tandem mass spectrometry to screen for autoreactive antibodies against 13,028 antigens extracted from 46 human tissue types and 7 biological fluids.^22^ 64 samples from 30 stage III-IV melanoma patients across three patient visits (pretreatment, timepoint A, timepoint B) were analyzed for autoreactivity (online supplemental table 4).

## Results

### Extrafollicular B cell differentiation, B regulatory induction and Th2-biased antibody isotypes in peripheral blood of patients with advanced melanoma

**Figure 1:**
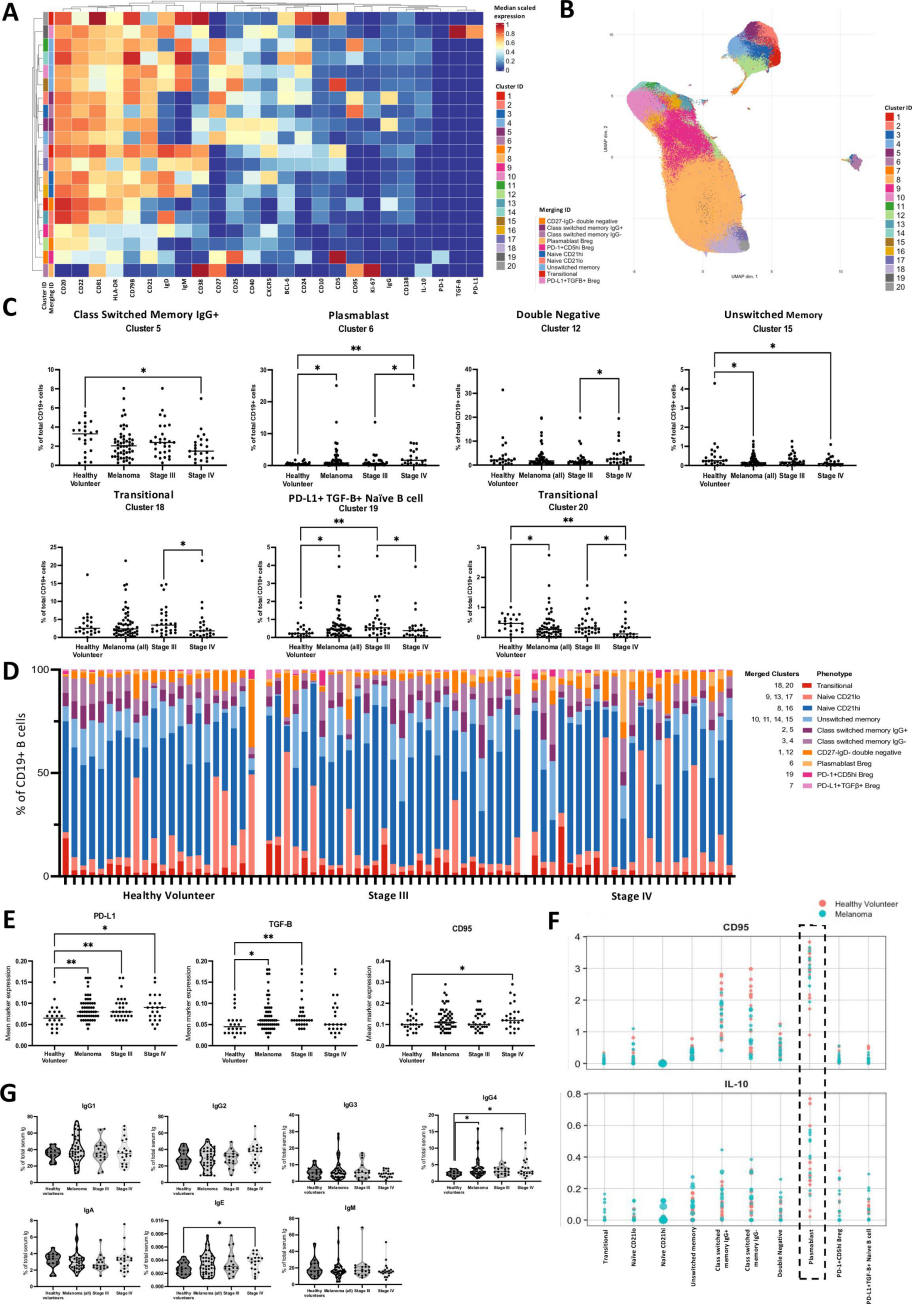
Peripheral blood B cells and antibodies exhibit regulatory features in patient with advanced stage III and IV melanoma versus healthy volunteer blood. Mass cytometry (CyTOF, 25 markers) analyses of B cell peripheral blood populations in treatment-naive advanced patients with stage III and IV melanoma and healthy volunteers (HVs) (total n=74; HVs n=22, patients with stage III/IV melanoma n=52) (**A–F**). (**A–B**) Heatmap showing normalized median scaled expression of B cell markers (X axis) within 20 unsupervised clusters (Y axis), generated by the FlowSOM algorithm (**A**); and 20 B cell clusters visualized in UMAP plot (**B**). (**C**) Comparison of the relative abundance of differentially expressed unsupervised B cell clusters in HV and patients with melanoma stratified by stage of disease. (**D**) The 20 B cell clusters identified in (**A**) were merged into 10 defined B cell clusters and visualized in a bar chart showing the relative proportion of merged clusters per sample. (**E**) Mean scaled expression of PD-L1, TGFβ and CD95 in B cells from HVs and patients with melanoma stratified by stage. (**F**) In-depth review of marker expression per B cell population illustrating high IL-10 and CD95 in the plasmablast population. (**G**) Violin plots comparing serum antibody isotype titres (Luminex multiplex immunoassay) in HVs and patients with melanoma stratified by stage, showing enhanced levels of IgG4 in patient blood, and in stage IV sera compared with HV sera (Mann-Whitney U test) (based on non-parametric distribution). *p<0.05; **p<0.01. Breg, regulatory B cell; IL-10, Interleukin-10; PD-1, Programmed cell death protein 1; PD-L1, Programmed death ligand 1; TGFβ, Transforming Growth Factorβ; UMAP, Uniform Manifold Approximation and Projection.

**Figure 2:**
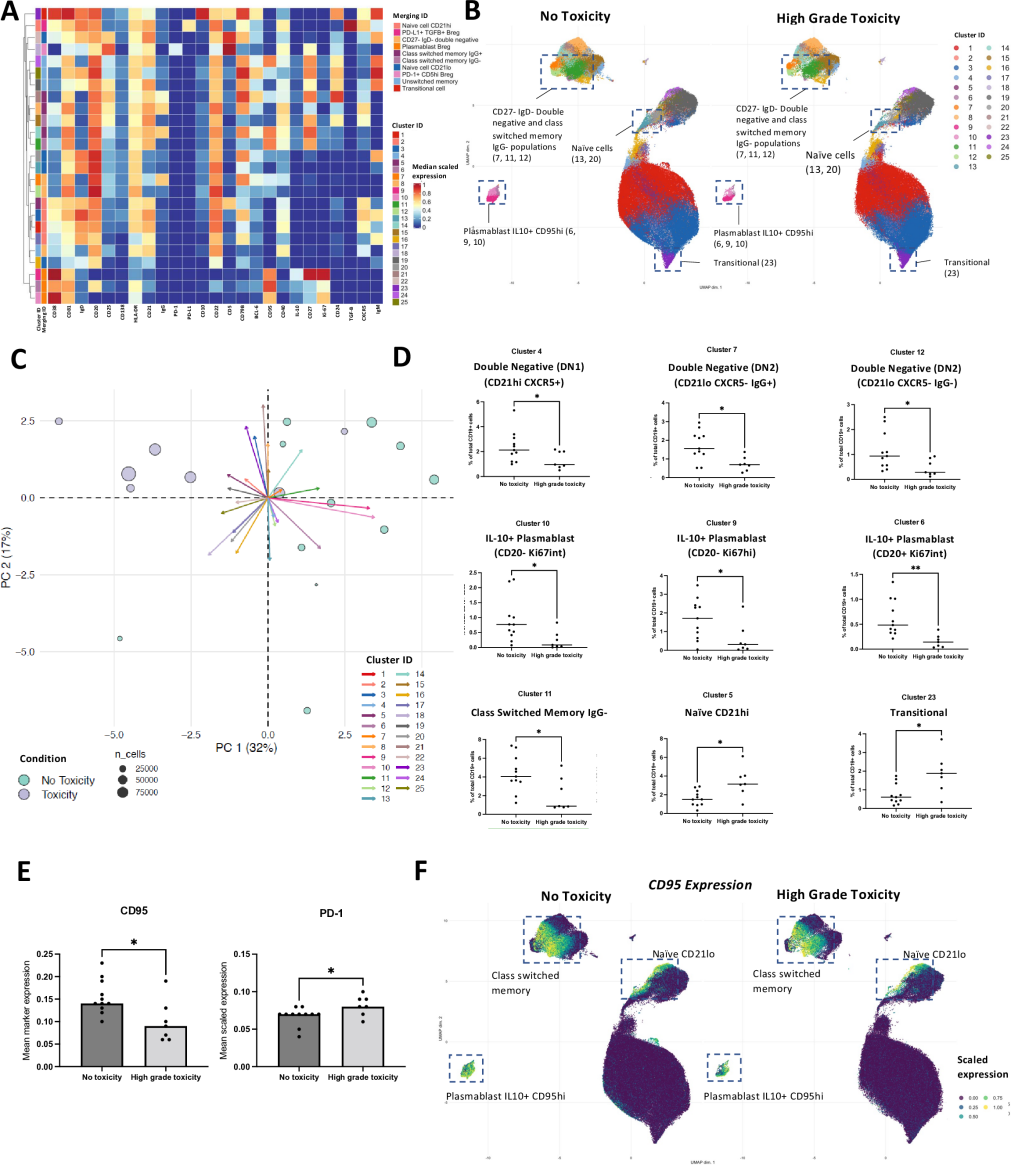
Baseline B cell phenotypes predict immune-related anti-PD-1-related adverse events. CyTOF (25 markers) analyses of B cell peripheral blood populations in treatment-naive advanced patients with stage III and IV melanoma (total n=18: no toxicity n=11, high-grade (grade 3 and above) toxicity n=7). (**A**) Heatmap showing normalized median scaled expression of B cell markers (X-axis) within 25 unsupervised B cell clusters (Y-axis) generated with the FlowSOM algorithm. (**B**) Comparative UMAPs of B cell phenotypes in patients with melanoma stratified by onset of “no toxicity” versus “high-grade toxicity” to checkpoint blockade. Clusters highlighted by boxes relate to clusters 4, 5, 6, 7, 9, 10, 11, 12 and 23 identified by FlowSOM clustering in (**A**). All samples are randomly down-sampled to 30,000 B cells/sample. (**C**) Principal Component (PC) dimensionality reduction plot illustrating centered log-ratios (CLRs) of proportions of B cell clusters across samples of patient with melanoma taken prior to anti-PD-1 treatment. Clusters described in (**A**) identify CLRs of cluster proportions across samples belonging to two cohorts: patients with melanoma who experience “no toxicity” to anti-PD-1 versus those that experience “high-grade toxicity”. PC1 largely separates the two cohorts. (**D**) Relative abundance of unsupervised B cell clusters that are statistically significantly differentially expressed between “no toxicity” and “high-grade toxicity” groups at baseline. Plasmablast Breg and double negative (DN) clusters were enriched at baseline in the no toxicity patients. *p<0.05; **p<0.01. (**E**) Comparisons of mean scaled expression of B cell markers in conditions of “no toxicity” versus “high-grade toxicity” reveal the Breg-associated and exhaustion marker CD95 is enriched in the no toxicity group at baseline, while PD-1 is enriched in the high-grade toxicity cohort. *p<0.05. (**F**) UMAPs comparing the expression of exhaustion marker CD95 across various B cell clusters between “no toxicity” and “high-grade toxicity” patients. Breg, regulatory B cell; CyTOF, cytometry by time of flight; IL-10, Interleukin-10; PC, Principal Component; PD-1, Programmed cell death protein 1; PD-L1, Programmed death ligand 1; TGFβ, Transforming Growth Factorβ; UMAP, Uniform Manifold Approximation and Projection.

We evaluated the circulating B cell compartment in patients with stage III/IV melanoma (n=52) and healthy volunteers (HV) (n=22) (online supplemental tables 1 and 2) by mass cytometry (CyTOF, 25 B cell lineage markers) (online supplemental table 3 and figure 2). We applied an unsupervised FlowSOM clustering algorithm, identifying 20 B cell populations at various maturation stages (figure 1A,B). The relative abundance of CD19+of total CD45+populations, in HV and in melanoma groups when stratified by disease stage were comparable (online supplemental figure 3A). Compared with HV, patients had lower frequencies of class-switched IgG+memory B cells (Cluster 5), and unswitched memory B cells (Cluster 15) and higher frequencies of IL-10+plasmablasts (Cluster 6) (figure 1C). Stage IV melanoma was associated with increased double-negative (DN) B cell (Cluster 12) and plasmablast (Cluster 6) frequencies compared with stage III melanoma, pointing to regulatory and extrafollicular maturation in more advanced disease (figure 1C). Compared with HV, patients showed higher PD-L1+TGFβ+ naive B cell levels, driven by the stage III cohort (Cluster 19).

To investigate whether changes in the B cell compartment could be observed in the overall population, manual merging of clusters into ten canonical B cell subsets was conducted as before.^17 23^ Six clusters were perturbed in melanoma, largely reflecting differences in unmerged cluster analyses (figure 1D, online supplemental figure 3B). Advanced melanoma showed reduced class-switched IgG+memory and transitional B cell subsets, enhanced plasmablasts, CD21lo naïve and DN populations, driven by stage IV disease and PD-L1+TGFβ+ naive B cells, driven by stage III (online supplemental figure 3B).

Evaluating overall marker expression, PD-L1, TGFβ and CD95 were upregulated on B cells from patients versus HV, indicative of expanded B cells with regulatory properties (figure 1E). When mean marker expression was compared on B cell subsets in HV and patients, the highest expression of IL-10 and CD95 was observed on circulating plasmablasts, found at higher frequencies in patient circulation (figure 1F).

Analysis of antibody isotype distribution in sera of CPI-naïve patients (n=38) and in patients with stage IV disease (n=20) showed significant enrichment for IgG4 (% of total Ig) compared with HV (n=16) (figure 1G) consistent with literature,^9^,^1224^ and IgE enrichment in stage IV disease compared with HV.

Hence, in patients we detected the expansion of B cells expressing regulatory markers PD-L1, TGFβ and CD95 alongside IL-10+ plasmablasts, indicative of enhanced regulatory responses. Alongside, antibody isotypes were skewed towards IgG4 and IgE, denoting prominent Th2-biased humoral responses in advanced melanoma.

### Baseline IL-10+ plasmablasts and DN B cells predict lack of irAEs due to anti-PD-1 therapy

We next investigated pretreatment B cell subset frequency and phenotype in patients with stage III/IV melanoma (n=18) who subsequently developed high irAEs (n=7) to CPI and those with no toxicity (n=11) (CyTOF) as above (figure 2A,B, online supplemental table 3). While no differences in CD19+B (% of total CD45+) cell abundance were seen in patients stratified by toxicity (online supplemental figure 4A), UMAP (Uniform Manifold Approximation and Projection) plots (figure 2B) demonstrated visual enrichment in several populations in the no toxicity cohort. High-dimensionality reduction PC (Principle Component) plot based on centered log-ratios (figure 2C) of sample proportions across clusters showed separation between no toxicity versus high-grade toxicity patients across PC1 (Principle Component 1).

Dot plots of cluster abundance confirmed seven clusters as significantly more abundant in the “no toxicity” patients at baseline: IL-10+plasmablast populations (Clusters 6, 9, 10); DN IgD-CD27- populations (Clusters 4, 7, 12); and an alternatively class-switched IgG- memory B cell population expressing IL-10 and CD95 (Cluster 11). Conversely, in the high-grade toxicity cohort at baseline, there was enrichment of naïve CD21hi (Cluster 5) and transitional (Cluster 23) populations. Of the DN B cell clusters with greater frequency in the no toxicity cohort, two had a CD21loCXCR5 phenotype akin to DN2 cells, a hallmark of extrafollicular B cell responses seen in autoimmune diseases such as systemic lupus erythematosus (SLE)^25^ (figure 2D). All three IL-10+plasmablast populations were identified with variable expression of CD20 and Ki67 and observed at higher frequency in the no toxicity cohort. These observations were confirmed on clusters manually merged to represent canonical B cell subsets (online supplemental figure 4B,C). No significant association between irAE development and overall survival was seen (n=13) (online supplemental figure 4D). Furthermore, in an independent cohort of 14 patients with advanced solid tumors, lower levels of baseline CD19+IL-10+B cells were found in individuals who subsequently experienced high-grade (n=7) irAEs to checkpoint inhibitor immunotherapy (online supplemental figure 5 and table 5). These data suggest that IL-10 expression in total patient B cells can also be detected following ex vivo stimulation and confirm that higher IL-10+B cells at baseline prior to immunotherapy predict lower irAEs in an independent metastatic disease cohort.

Analysis of mean marker expression on all B cells showed differential expression of regulatory markers CD95 and PD-1 in “high-grade toxicity” versus “no toxicity” cohorts (figure 2E,F). CD95, observed at highest levels on plasmablasts, CD21lo naïve and DN B cells, was enhanced in the “no toxicity” cohort at baseline. Overall PD-1+B cell expression was significantly higher in the group that subsequently developed “high-grade toxicity”, a phenomenon relevant in CPI therapy as these drugs may also directly modulate PD-1+B cells.

Furthermore, we performed univariate and pairwise-adjusted logistic regression for each immune feature, adjusting individually for age, sex or stage. The calculated ORs showed minimal change when adjusted for covariates, suggesting that the observed associations with irAEs are independent of demographic or tumor characteristics (online supplemental table 6). Overall, increased baseline frequencies of B cell subsets, associated with extrafollicular differentiation and regulatory functions, may confer protection against high-grade irAE development.

### Non-classical isotypes and autoreactive antibodies at baseline predict lack of irAEs to anti-PD-1

**Figure 3:**
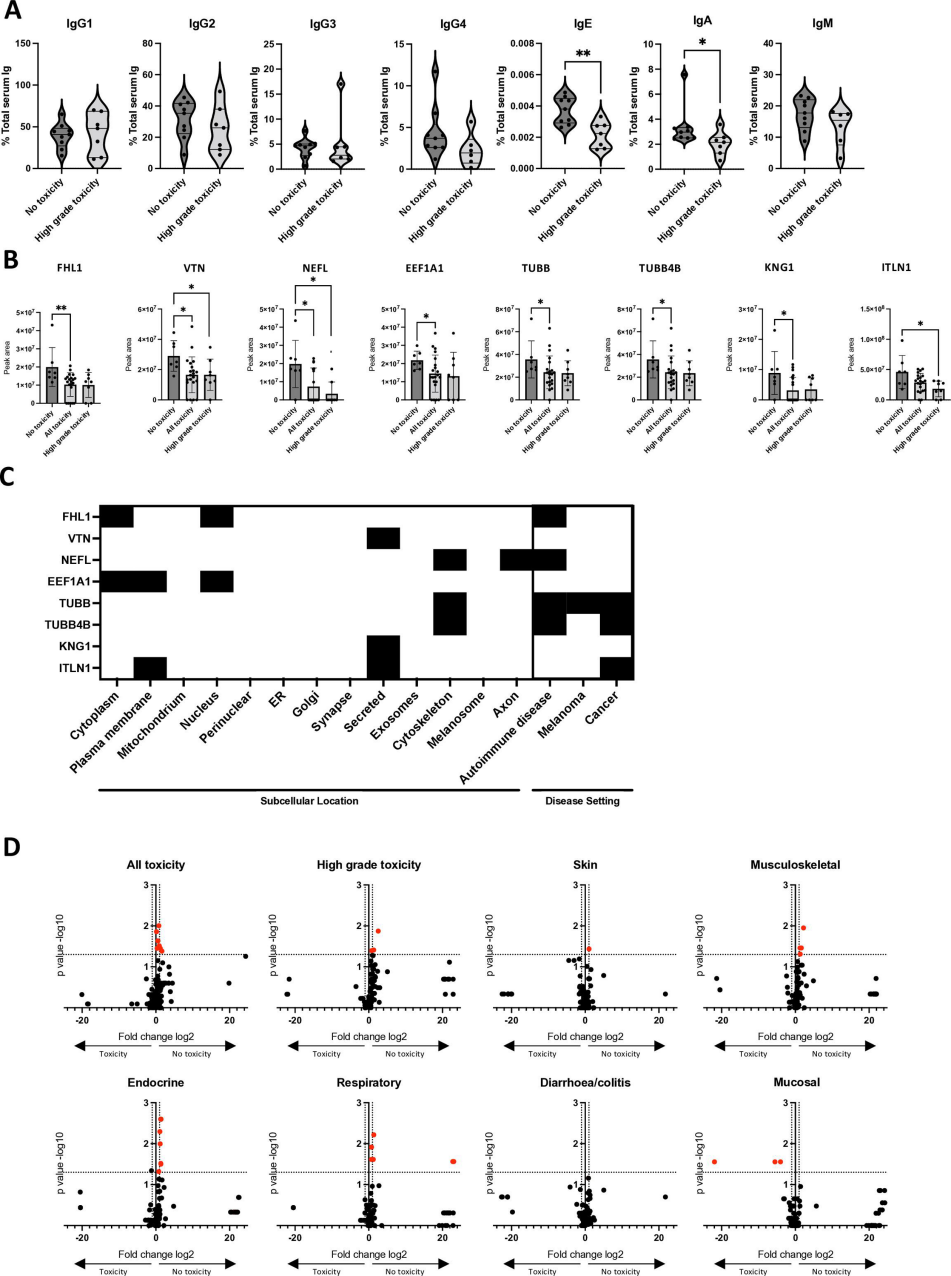
Non-classical isotypes and autoreactive antibodies at baseline predict lack of toxicity to anti-PD-1. (**A**) Violin plots comparing pretreatment IgG1, IgG2, IgG3, IgG4, IgM, IgE, IgA titres in serum of patient with melanoma (n=18) as a proportion of total immunoglobulin concentration (Luminex multi-plex immunoassay). Patients were stratified according to those who subsequently did not develop toxicity (no toxicity, n=11) to checkpoint inhibitor and those with high-grade (grade 3 and above) toxicity (n=7). Statistical analysis performed using Mann-Whitney U test. *p<0.05; **p<0.01. (**B**) Immuno-mass spectrometry (IMS) of serum immunoglobulins (IgG) revealed higher baseline autoreactivity in patients with melanoma treated with anti-PD-1 checkpoint blockade who did not develop immune-related toxicity. Proteins pulled down by antibodies in patient serum that are statistically significantly different in the presence or absence of toxicity as quantified by IMS peak area. *p<0.05. (**C**) Heatmaps of antigen cellular/extracellular location and summary of known relevance of autoantigen in disease settings. (**D**) Volcano plots summarizing differential putative autoantibody states in no toxicity versus toxicity states, stratified by organ system. Each dot represents a protein target. The Y axis indicates fold-change (FC) between “no toxicity” (positive FC values) and “toxicity” (negative FC values). Dots above the horizontal dotted line indicate significant difference in peak areas in the cohorts (p<0.05), highlighted as red dots. PD-1, Programmed cell death protein 1.

irAEs can mimic inflammatory and autoimmune disease and antibody isotypes may be highly relevant to their pathogenesis. In a subset of patients for whom serum was available, we evaluated serum antibody isotypes (IgG1, IgG2, IgG3, IgG4, IgE, IgA, IgM) at pretreatment: patients were stratified into “no toxicity” (n=11) versus “high-grade toxicity” (n=7) to CPI (figure 3A). Baseline enrichment with IgA (p=0.0311) and IgE (p=0.0052) was protective against irAE development (figure 3A). Univariate and pairwise-adjusted logistic regression for antibody isotype (adjusting individually for age, sex, or stage) of calculated ORs showed minimal change when adjusted for covariates, suggesting that associations with toxicity are independent of demographic or tumor characteristics (online supplemental table 7).

Autoantibodies targeting self-antigens are markers for several autoimmune diseases including connective tissue diseases such as SLE and rheumatoid arthritis (RA), and endocrinopathies such as hypothyroidism. They may develop prior to SLE/RA clinical manifestations, and anti-nuclear antibodies may be present in up to 25% of seemingly healthy individuals.^26^ Linking autoantibodies to CPI-related irAE yielded conflicting results.^27^,^30^ We investigated baseline autoreactivity, using IMS, taking a proteome-wide approach for autoantigen capture, screening serum IgG antibodies against 13,028 candidate proteins from a range of tissue extracts and biological fluids (online supplemental table 4). Patients were classified as “no toxicity” (n=7), “high-grade toxicity” (n=8), “all toxicity” (n=21) and further stratified according to organ system affected by toxicity: “skin” (n=5), “musculoskeletal” (n=11), “endocrine” (n=5), “respiratory” (n=3), “diarrhoea/colitis” (n=10), and “mucosal” (n=3). This classification was selected due to the potential organ-specific nature of autoantibodies and the premise that “non-severe” toxicities such as hypothyroidism may be autoantibody-mediated. Baseline antibody reactivity to several candidate autoantigens (FHL1, VTN, NEFL, EEF1A1, TUBB, TUBB4B, KNG1, ITLN1) previously reported autoantibody targets in melanoma, cancer, and autoimmune diseases^31^,^33^ was significantly increased in the “no toxicity” patient cohort compared with the “high toxicity” and “all toxicity” groups (figure 3B,C). Comparison of each peptide’s signal peak areas (heatmap of baseline serum samples) showed no clear autoantigen targets segregation (online supplemental figure 6).

To obtain an overview of differential autoantibody expression in relation to organ-specific toxicity, volcano plots were generated summarizing all protein targets identified, each dot representing a protein target and the presence of a putative IgG autoantibody. Fold-change comparison (X axis, figure 3D) was plotted for no toxicity (positive fold-change) versus toxicity (negative fold-change). Peak area signals that were significantly different between no toxicity and toxicity states were indicated by red dots. Overall, patients who did not experience toxicity had higher baseline autoantibody signal intensity. Most significantly increased signal intensities were observed in the “no toxicity” group. As the only exception, three putative autoantibodies were significantly higher in “mucosal toxicity” compared with “no toxicity” (PCBP2, COL6A1, SEMG1), referring to patients developing oral mucositis. COL6A1 (Collagen Type VI Alpha 1 Chain) is a component of the extracellular matrix found in oral mucosa and several other tissues and may therefore be clinically relevant to this toxicity, but has not been previously reported in mucositis or autoimmune disease.

These findings, in line with data from a previously published study,^30^ point to higher baseline autoreactivity in patients who do not subsequently develop toxicity to CPI.

### Anti-PD-1 induces B cell subsets associated with regulatory responses but skews circulating antibody profiles towards pro-inflammatory isotypes

We characterized changes in circulating B cell profiles in longitudinal samples of patients with stage III/IV melanoma treated with CPI, at three timepoints: baseline pretreatment (n=32); timepoint A (after 6–8 weeks, n=31) and timepoint B (after 12 weeks, n=15) on-treatment (online supplemental figure 1).

**Figure 4:**
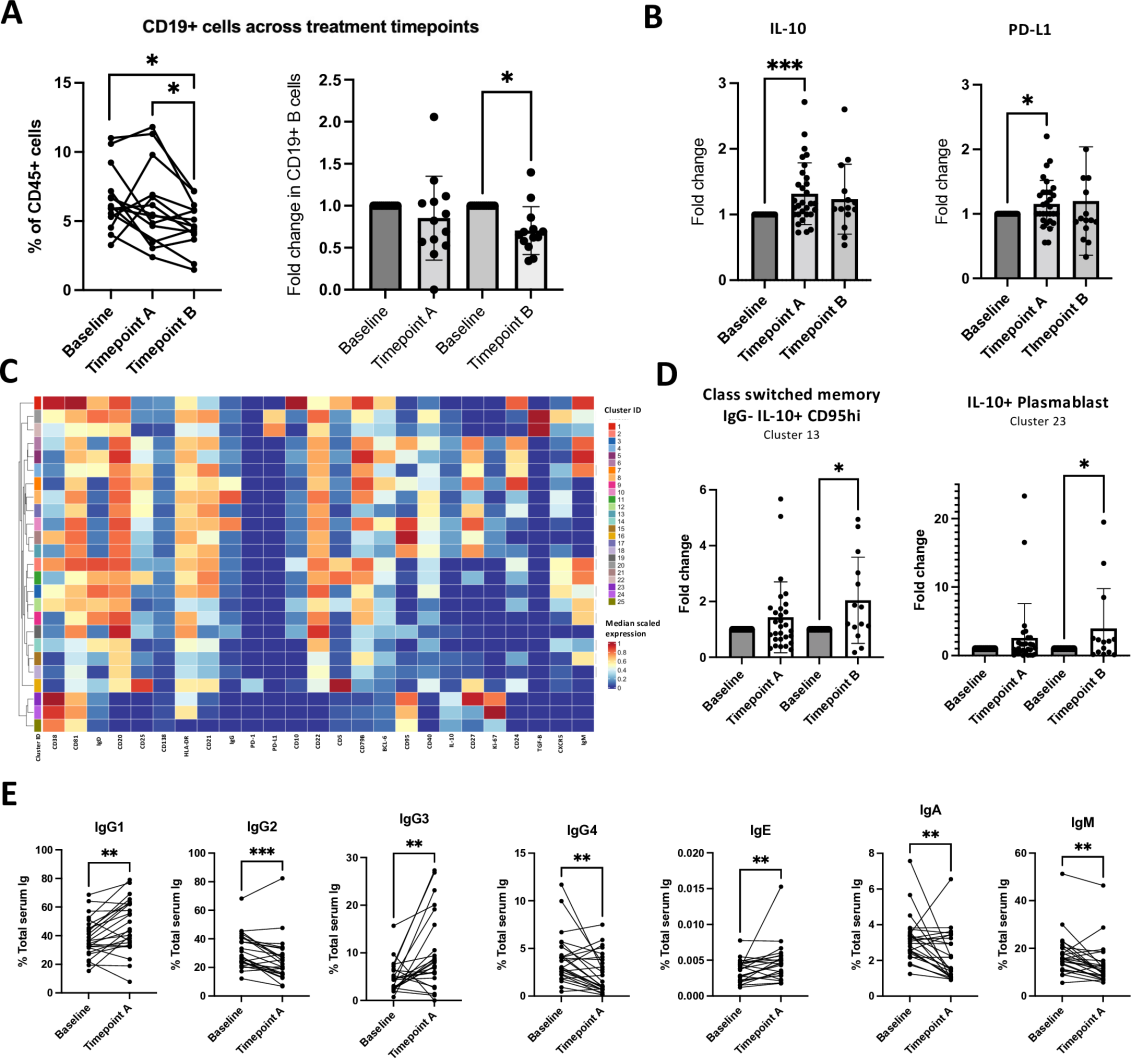
Anti-PD-1 therapy enhances memory B cells and plasmablast-like populations and alters the profile of circulating antibody isotypes on treatment. (**A**) Decrease in B cells seen after 12 weeks of treatment with anti-PD-1 therapy in stage III/IV melanoma. Paired analysis (left) and fold-change (right) comparison of B cells at baseline versus timepoint A versus timepoint B after 12 weeks of treatment (baseline pretreatment (n=32); timepoint A (after 6–8 weeks on-treatment, n=31) and timepoint B (after 12 weeks on-treatment, n=15) (*p<0.05). (**B**) Regulatory IL-10 and PD-L1 marker expression was elevated on-treatment with anti-PD-1 therapy in a cohort of patients with melanoma (n=32 at baseline, n=31 timepoint A, n=15 timepoint B). Mean scaled expression of each marker was extracted using the CATALYST pathway; graphs depict fold-change from baseline for timepoints A and B. *p<0.05, ***p<0.001. (**C**) Peripheral blood B cell phenotyping at baseline and on-treatment with anti-PD-1 therapy (n=78 samples). Heatmap showing normalized median scaled expression of B cell markers (X-axis) within 25 B cell clusters (Y-axis) generated with FlowSOM algorithm. (**D**) Two B cell subpopulations (identified using FlowSOM clustering algorithm (**C)**) increased on-treatment: class-switched memory IgG-IL-10+CD95hi (Cluster 13, p=0.035); and plasmablast-like IL-10+CD95 hi population (Cluster 23, p=0.042) (paired analysis performed using Wilcoxon rank test, *p<0.05). (**E**) Paired statistical comparison of circulating antibody isotypes was performed comparing fold-change on-treatment and antibody isotype as percentage of total circulating antibody titer at timepoint A on-treatment. Statistical analysis using the Wilcoxon paired rank test. *p<0.05, **p<0.01, ***p<0.001. IL-10, Interleukin-10; PD-1, Programmed cell death protein 1; PD-L1, Programmed death ligand 1.

A significant decrease in CD19+B cells (% of CD45+cells) in the whole cohort and in stage III disease was observed at timepoint B compared with baseline (figure 4A; online supplemental figure 7; absolute %, Wilcoxon rank test, p=0.01; mean fold-change 0.77, Wilcoxon rank test, p=0.01). A decline in circulating B cells was associated with earlier onset of irAE (online supplemental figure 8A). The median time to toxicity was 65 days and to severe toxicity 93 days, and blood samples were taken~56 days after treatment initiation, suggesting that B cell decrease may predate irAE onset. Furthermore, a~13% decrease or greater in B cells early on treatment was associated with poorer overall survival (online supplemental figure 8B). These further highlight the importance of B cell densities in the context of anti-PD-1 treatment toxicity and treatment response.

IL-10 (fold-change 1.32, p=0.0013) and PD-L1 (fold-change 1.15, p=0.028) B cell expression was upregulated after 6–8 weeks (figure 4B). In pooled samples from all timepoints (n=78, to allow like-for-like comparison of cluster abundance in each patient across different timepoints), circulating B cell subpopulations like those described at baseline were identified by the FlowSOM clustering algorithm to CD19+ cells (figure 4C). In these longitudinal samples, we conducted paired analysis of B cell cluster abundance (Wilcoxon rank test) to investigate whether CPI alters phenotype distribution. At timepoint B, two subpopulations were significantly enriched: Cluster 23 (IL-10+CD95hi plasmablasts, fold-change 3.94, p=0.041) and Cluster 13 (IgG-IL-10+CD95hi, class-switched memory B cell fold-change 2.04, p=0.035) (figure 4D). This suggests that CPI promotes populations of class-switched, antibody-producing cells although with a regulatory phenotype.

Diverse functionality is attributed to different antibody isotypes and therefore the balance of circulating isotypes may be directly relevant to treatment outcomes. We therefore investigated whether antibody isotype distribution is influenced by CPI (baseline, timepoint A (n=25), and timepoint B (n=11)) (figure 4E). Proportional increases in serum titers were observed at timepoint A for: IgG1 (mean fold-change 1.27, p=0.0051), IgG3 (mean fold-change 2.92, p=0.0066) and IgE (mean fold-change 1.47, p=0.0011). Decreases are observed at timepoint A in: IgG2 (mean fold-change 0.78, p=0.0006), IgG4 (mean fold-change 0.67, p=0.0061), IgA (mean fold-change 0.74, p=0.0004), IgM (mean fold-change 0.79, p=0.0088). Serum antibody isotype levels were not significantly altered at timepoint B except for IgG4, which increased from baseline (online supplemental figure 9).

These results highlight the circulating humoral compartment as a dynamic part of CPI response, pointing to humoral response rebalance towards class-switched, antibody-producing cells, and consistent with a temporal shift in favor of classical immunostimulatory isotypes IgG1, IgG3 and away from less immunoactive IgG2, and regulatory IgG4.

### CPI therapy is associated with heterogeneous changes in serum autoantibodies

**Figure 5:**
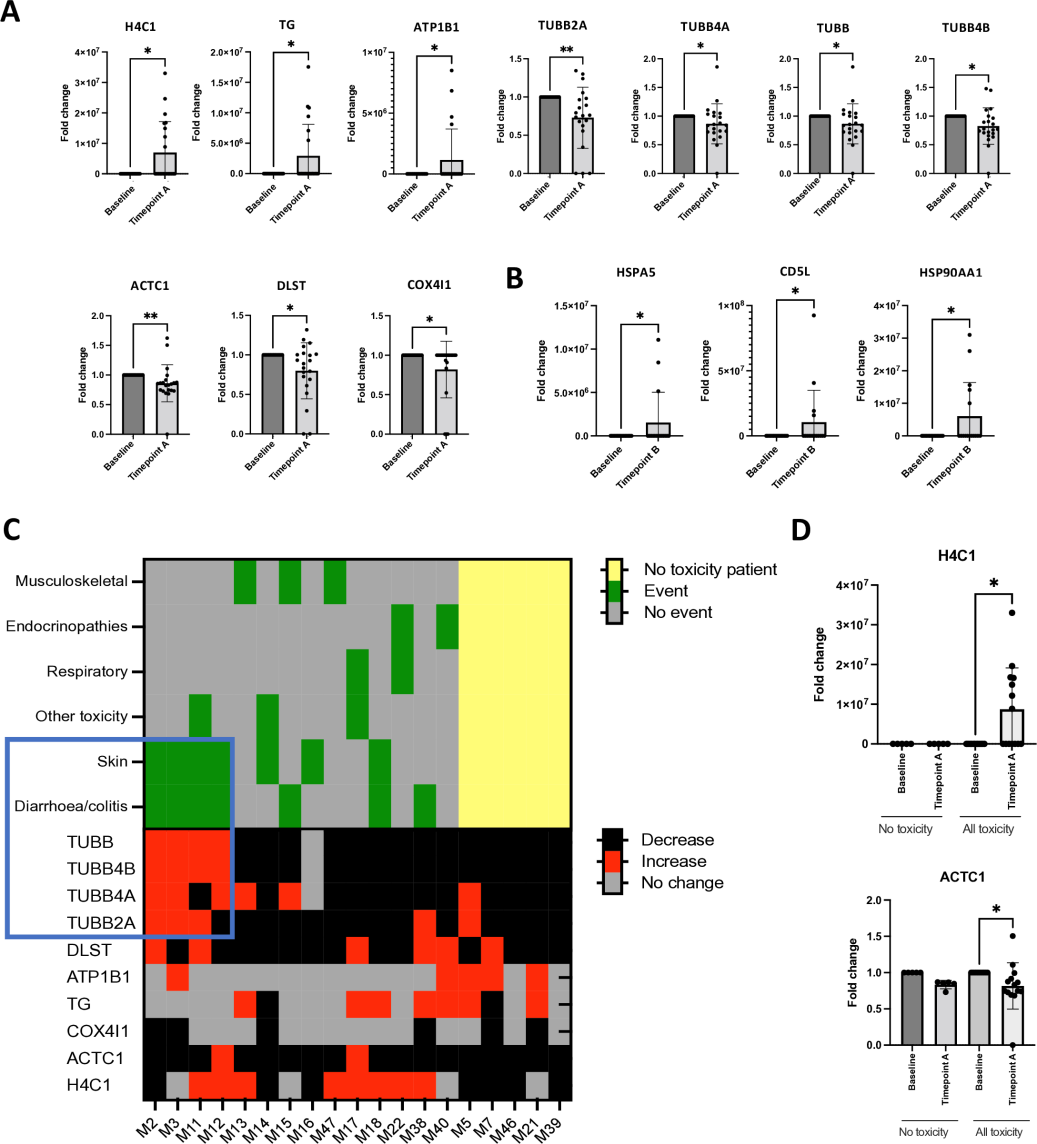
Anti-PD-1 therapy alters serum autoreactivity in melanoma. Autoantibodies to putative antigens were identified using immuno-mass spectrometry (IMS) screening of patient serum against 13,028 human proteins. (**A–B**) IMS of serum immunoglobulins (IgG) (proteins pulled down by IgG antibodies in patient serum indicative of the presence of autoantibodies) revealed shifts in autoreactivity in melanoma patients treated with anti-PD-1 therapy. Fold change in peak area at baseline compared with on-treatment serum samples was evaluated for candidate proteins. Statistically significant fold changes are illustrated at timepoint A (**A**) and timepoint B (**B**). At timepoint A (**A**), significant increases in putative autoantibody titres to three proteins H4C1, TG and AT1B1, and decreases in autoantibody titres to three antigens, including four tubulin proteins (TUBB2A, TUBB4A, TUBB, TUBB4B, ACTC1, DLST and COX4I1), were detected. At timepoint B (**B**), autoantibodies were increased for three proteins, HSPA5, CD5L and HSP90AA1. *p<0.05, **p<0.01. (**C**) Heatmaps of serum autoreactivity and immune toxicity to anti-PD-1 shown for each patient. Putative autoantibodies that show a significant fold change in peak area (from A) were analyzed in relation to organ-specific toxicity. Top heatmap depicts the incidence of organ-specific toxicity (yellow: patients who did not experience toxicity; green: specific toxicity event; gray: no specific toxicity event). Bottom heatmap illustrates whether fold change of each protein is increasing (red), decreasing (black) or not changing (gray) on treatment. Each column represents an individual patient. (**D**) Fold change in putative autoantibodies is stratified by the presence or absence of immune toxicity on treatment. Autoreactivity against H4C1 was differentially increased in patients who experienced toxicity compared with those who do not develop toxicity. Wilcoxon signed rank test. *p<0.05. PD-1, Programmed cell death protein 1.

The PD-1 pathway can negatively regulate B cell function and contribute to suppression of aberrant self-reactive B cells and subsequent autoreactivity.^34^ Inhibition of PD-1 through CPI may therefore have direct or indirect effects on B cell autoreactivity.^27 28^ We investigated changes in putative autoantibodies during treatment using IMS against 13,028 candidate human proteins, calculating fold-change in peak area output at baseline and on-treatment for each protein target (figure 5A,B). We found significantly increased autoantibody titers to three putative antigens (H4C1, TG, AT1B1), and significantly decreased autoantibody titers to seven antigens including four tubulin proteins (TUBB2A, TUBB4A, TUBB, TUBB4B, ACTC1, DLST, COX4I1) at timepoint A; and increased autoreactivity to three putative autoantigens (HSPA5, CD5L, HSP90AA1) at timepoint B.

We then stratified treated patients based on the presence of organ-specific toxicity, the overall presence or absence of toxicity (figure 5C) and evaluated changes in the significant putative autoantibodies. Sera from four patients who developed both skin toxicity and colitis/diarrhea also showed increased autoantibodies to tubulins (TUBB2A, TUBB4A, TUBB, TUBB4B) on treatment. Autoreactivity against H4C1, a histone and recognized target of autoreactivity in drug-induced lupus, increased on-treatment exclusively in patients who developed toxicity (figure 5C,D). Interestingly, increased fold change in autoantibodies against TG (thyroglobulin) was detected on treatment in both toxicity and no toxicity groups and did not correlate with the onset of endocrine toxicity.

Modulation of autoreactivity on treatment may reflect dynamic changes in B cell antigenic responses, potentially arising from combined immune activation via CPI, and humoral autoreactive responses to tissue damage and subsequent antigen release. Despite no correlation between immune toxicity and organ-specific autoantibody responses, increased anti-histone (H4C1) antibodies on treatment were detected in patients who developed toxicity.

### Enriched circulating naïve, transitional, regulatory B cell subsets and alternative antibody isotypes at baseline predict less favorable overall survival in anti-PD-1 therapy

**Figure 6:**
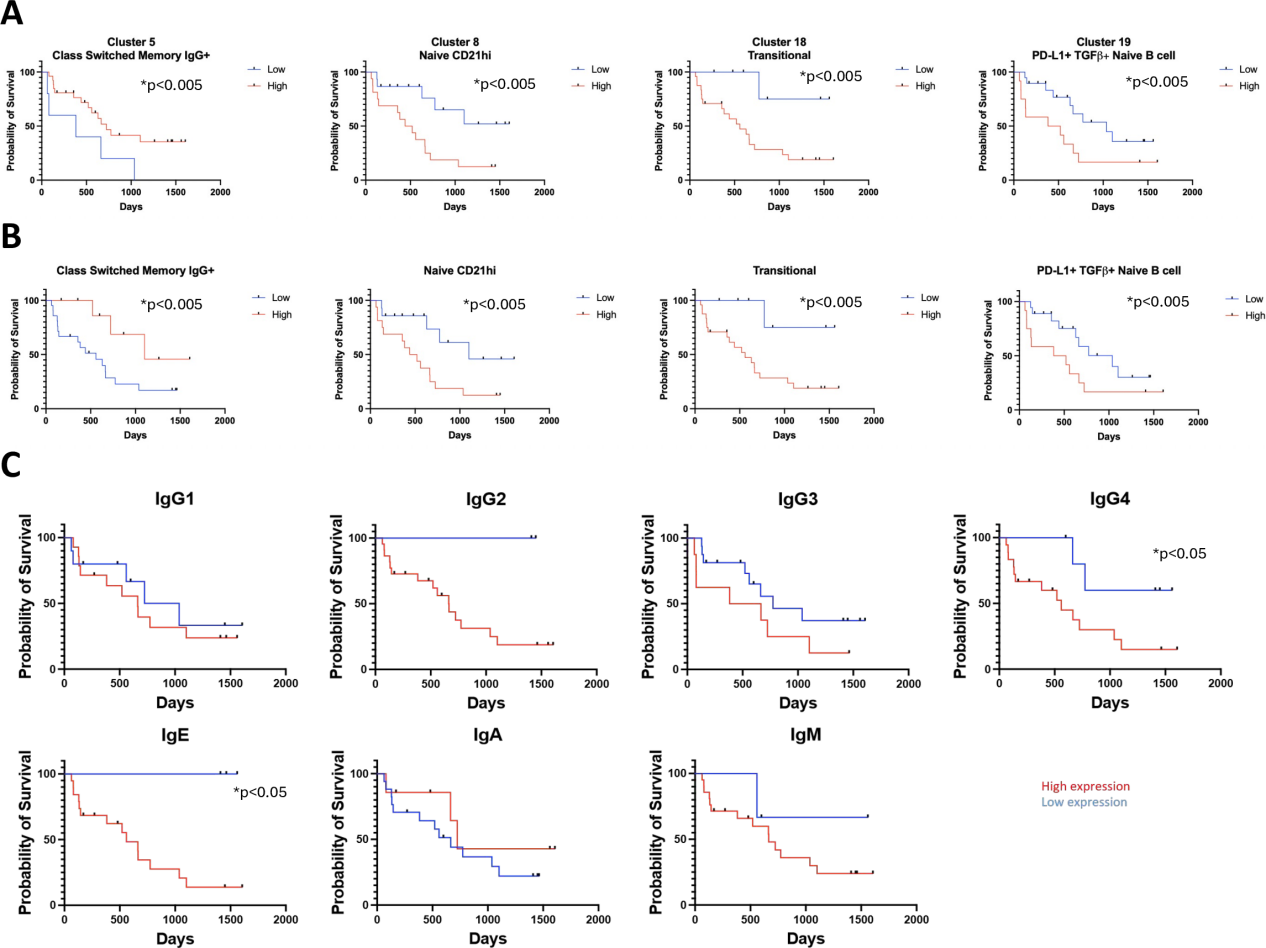
Regulatory B cell subsets and antibodies predict lower overall survival in patients treated with anti-PD-1 therapy. (**A**) Kaplan-Meier curves for B cell phenotypes that significantly predict overall survival, extracted from unsupervised clusters identified in figure 1. *p<0.05, Gehan-Breslow-Wilcoxon statistical test. (**B**) Kaplan-Meier curves for B cell phenotypes that significantly predict overall survival, extracted from merged B cell clusters identified in figure 1. *p<0.05, Gehan-Breslow-Wilcoxon statistical test. (**C**) Kaplan-Meier curves illustrating correlation between antibody isotype and overall survival. Relative enrichment of serum IgG4 and IgE in patients with melanoma prior to checkpoint immunotherapy treatment was negatively associated with overall survival in those treated with anti-PD-1 therapy. IL-10, Interleukin-10; PD-1, Programmed cell death protein 1; PD-L1, Programmed death ligand 1.

We investigated whether pretreatment B cell subset frequency may impact overall survival in CPI-treated patients. B cell clusters in figure 1A–C and only patients with detectable disease (n=21) at baseline were used for the survival analysis (median follow-up: 632 days; median time on-treatment: 210 days). Baseline increased frequency of class-switched IgG+memory B cells (Cluster 5) was associated with improved survival. Conversely, higher frequencies of transitional, CD21hi naïve and PD-L1+TGFβ+ naïve B cells (Clusters 8, 18, 19) were associated with worse survival (figure 6A). These associations were also seen in manually-merged clusters to create canonical B cell subsets (figure 6B). Furthermore, baseline relative enrichment of IgG4 and IgE was negatively associated with overall survival in CPI-treated patients (log rank test, p=0.046; figure 6C).

Consistent with reports of disparate B cell differentiation states between blood and tumors,^7 17 35^ scRNAseq (single-cell RNA sequencing) analyses of melanoma lesion samples^36^ revealed B cells and plasma cells pre-CPI and post-CPI with class-switched phenotypes, and expressing CR2/CD21, CD95/Fas and TGFβ, while few cells expressed PD-L1 and IL-10. In samples post-CPI, cells from responders, compared with non-responders, showed higher IgHA1 (plasma cells) and IgHA2 (B cells and plasma cells) expression and lower IgHG1-4 (plasma cells) expression (online supplemental figure 10).

Prior to CPI, increased frequencies of circulating class-switched memory B cells, likely pointing to a mature adaptive immune response, predicted better survival. Inverse correlation between survival and frequency of transitional, CD21hi naïve and PD-L1+TGFβ+ B cells may relate to immature and enhanced regulatory responses, denoting lower immune fitness that could limit antitumor immunity. In tumors, enhanced IgA-expressing plasma cells post-treatment were associated with response.

## Discussion

Identifying patients who may develop severe immune side effects and those likely to benefit from CPI therapy are important unmet needs in the clinical management of melanoma and will continue to pose a challenge with broadening use of CPI in the adjuvant setting. In contrast with tumor-infiltrating B cells, circulating B cells and antibodies may represent a more readily accessible and quantifiable tool. In this study, we identify shifts in B cell subsets, antibody isotypes and autoreactivity in advanced melanoma and provide evidence that these may predict toxicity during CPI and treatment efficacy in terms of survival.

The B cell compartment in advanced melanoma is distinct from that in healthy states and shows striking parallels with autoimmune and autoreactive states. Deep phenotypic analysis of circulating B cells from patients and HV demonstrated increased frequencies of DN B cells alongside IL-10+CD95+ plasmablasts, and concurrent collapse in memory B cell subsets in melanoma. These phenotypic shifts were enhanced in stage IV melanoma, suggesting that tumor progression and burden may be involved. Increased DN B cell frequencies may relate to enhanced regulatory responses, aberrant differentiation via extrafollicular maturation or immune exhaustion.^37 38^ Extrafollicular B cell maturation is implicated in the pathogenesis of autoimmune diseases such as SLE, in which these populations are predominantly driven by interferon-gamma.^25^ In such responses, activated naïve B cells undergo rapid differentiation into short-lived plasmablasts via CD27-IgD-CXCR5-CD21lo DN2 cells, which are encountered at low frequencies in health, but are grossly expanded in autoimmune responses.^39^ Extrafollicular B cell responses occur independently of germinal centers and therefore bypass immune checkpoints, resulting in increased autoreactive plasmablast frequencies.^40^ A drive towards Th2, especially alternatively activated Th2, responses is a prominent component of immune dysregulation in advanced melanoma, mimicking chronic inflammatory states. We observed increased circulating levels of Th2 classical (IgE) and Th2 alternative (IgG4) antibody isotypes, consistent with well-described Th2-associated cytokine profiles and dysregulated immune responses in advanced disease and indicate that tumor progression and burden could contribute to altered phenotypes. Together, these features point to a combination of autoimmune and alternatively-activated Th2 humoral response characteristics.

We found a decline in B cell frequencies after one cycle of anti-PD-1 treatment. Greater reduction in B cells early on treatment significantly correlated with shorter time to irAE onset, suggesting that B cell enrichment may be protective. In concordance, a previous report demonstrated B cell density decline following combination anti-CTLA-4 (Cytotoxic T-lymphocyte associated protein 4) and anti-PD-1 therapy^41^ and increased plasmablasts and CD21lo B cells.^41^ Our study focusing on anti-PD-1 monotherapy additionally shows prominent induction of regulatory B cells and plasmablasts in advanced melanoma and during anti-PD-1 therapy. We also observed dynamic shifts in antibody isotypes, namely increased frequency of “pro-inflammatory” IgG1 and IgG3 capable of complement fixation, and concurrent fall in IgG4 and IgM titers, early on-treatment, suggesting augmented class-switched B cell memory responses.

Increased baseline frequencies of several DN2 cells and plasmablast subsets, enrichment with IgA and IgE, and autoreactive antibodies directed against several candidate autoantigens were also seen in patients who did not subsequently develop irAEs. Extrafollicular B cell responses are features of autoreactivity. Our findings that increased baseline frequencies of subsets associated with extrafollicular differentiation are protective against irAE concur with enhanced serum autoreactivity observed in the non-toxicity patient cohort. This may suggest that irAE mechanisms may not be directly related to mounting autoantibody responses, consistent with a study reporting lower baseline autoantibody levels in patients who developed irAE; the authors proposed that a level of tolerance in patients with heightened baseline autoantibodies may be protective.^30^ Together, it is possible that antibody autoreactivity may not constitute a pathogenic mediator in irAE development but may signify a shift towards lower affinity antibodies featuring isotypes with lower potency in activating certain components of the immune response. In line, we report baseline enhanced DN B cell and plasmablast populations, enrichment of non-classical IgA and IgE isotypes and autoantibody profiles to certain autoantigens. While we could only screen for IgG serum autoreactivities, further insights may be derived from expanding proteomic screening to other antibody classes, IgA, IgE, IgM.

B regulatory cells are heterogeneous in phenotype but can inhibit pathogenic T cell expansion via IL-10 and TGFβ.^42^ B regulatory responses are defective in autoimmune disease such as SLE,^43^ but in melanoma TGFβ+PD-L1+ B cells are reported to promote FOXP3+ Treg induction.^7^ In concordance, upregulation of both TGFβ and PD-L1 on B cells in advanced melanoma and expansion of IL-10+plasmablasts and TGFβ+PD-L1+ naïve B cells indicate regulatory response induction. Plasmablasts were found to have the highest IL-10 expression of all subsets, further consolidating immune regulation roles. Expansion of IL-10+plasmablast subsets was also associated with irAE reduction, suggesting that regulatory responses are important to prevent inflammatory sequelae on treatment. Protective effects of IL-10+B cells are reported in other diseases such as allogeneic stem cell transplant recipients with chronic graft-versus-host disease.^44^ Our findings of IL-10+plasmablast deficiency in patients who subsequently develop irAEs are consistent with reported protective roles of these cells from immune-related inflammatory and autoimmune conditions.

Increased frequency of TGFβ+PD-L1+ naïve and transitional B cells at baseline predicts worse survival, while memory B cells associate with more favorable outcomes on CPI. This suggests that aspects of B regulatory responses may dampen antitumor immunity that would otherwise be enhanced on treatment. Transitional B cells have a high frequency of cells with B regulatory potential, and interestingly, this subset was similarly increased at baseline in those with poorer survival on therapy. Prominent increases in IL-10 and PD-L1 and induction of IL-10+class-switched memory B cells and IL-10+plasmablasts during treatment, however, suggest that CPI also induces regulatory responses. It therefore seems likely that for maximal treatment efficacy, a critical balance in such regulatory responses needs to exist, whereby toxicity is prevented but antitumor immune responses are simultaneously augmented. This concurs with reports that irAE onset may predict treatment responses and survival to CPI.^45^ Unlike circulating B cells, which feature diverse differentiation states, tumor-resident B cells are biased towards class-switched phenotypes.^17 35 46^ Post-CPI, IgA expression by tumor resident plasma cells associates with responders, denoting disparate tumor and peripheral B cell signatures and associations with CPI response.

Advanced melanoma may mimic chronic Th2-biased inflammatory states, consistent with serum enrichment of the immunologically “weak” IgG4, known for impaired immunostimulatory functions and for promoting propagation of melanoma and other cancers.^9^,^1247^ IgG4 is implicated in autoimmune processes and marks altered immune processes driven by prolonged antigen exposure, alternative Th2-biased immune environments and chronic inflammation. Of relevance, high IL-10 expressing plasmablasts may contribute to isotype switching which favors IgG4 in patients compared with healthy controls. In tumors, IL-10 can enhance IgG4 expression by B cells and, in the presence of IL-4, promote B cell class-switching to IgG4. Regulatory IL-10-expressing plasmablasts in melanoma may thus serve as one potential source of IL-10, contributing to impaired antitumor activity, in part by upregulating IgG4.

One hypothesis may be that irAEs represent “unmasking” of a predisposition to autoimmune phenomena, especially as some irAEs directly mimic traditional autoimmune conditions, for example, thyroid disorders. However, the irAE phenotype is broad and most toxicities do not represent disease states that classically correlate with organ-specific antibodies in the absence of CPI. We identify increased baseline IgG autoreactivity in those who did not subsequently develop irAE; on treatment, we find variable changes in autoreactive antibody titers but a general decline, especially in antitubulin antibodies. Autoreactive antibodies may reflect B cell phenotypes, namely plasmablasts and DN B cells. If developed via extrafollicular pathways, these cells may produce lower affinity antibodies that are not pathogenic, but rather markers of a rapid-responding, autoreactive humoral compartment. These may reflect a state of enhanced extrafollicular and regulatory response, and perhaps not always a predisposition to autoimmune disease.

Our study reveals distinct baseline circulating humoral profiles in advanced melanoma that may help predict toxicity and treatment responses to CPI. Uncovering these signatures harbors potential to aid patient selection a priori. Pretreatment B cell signatures may highlight patients that require closer monitoring for irAE or earlier escalation of treatment for irAE in those at risk of high-grade toxicity, for example, through early introduction of oral steroids or anti-TNF (Tumour Necrosis Factor) agents, to facilitate ongoing therapy and minimize treatment withdrawal. Modulated humoral responses on treatment illustrate that B cells are part of a dynamic immune response adjunct to checkpoint blockade and may contribute to the pathogenesis of treatment-associated toxicity and clinical outcomes. More broadly, if validated in larger cohorts, our findings may have relevance in informing the evolving clinical use of anti-PD-1 therapy, facilitate patient selection for treatment, guide therapeutic monitoring and early intervention of irAE.

## Supplementary material

10.1136/jitc-2025-011682 online supplemental file 1

## Data Availability

Data are available upon reasonable request.
