# Supplementary material for: Circulating immunoregulatory B cell and autoreactive antibody profiles predict lack of toxicity to anti-PD-1 checkpoint inhibitor treatment in advanced melanoma
Source: J Immunother Cancer. 2025 May 31;13(5):e011682. doi: 10.1136/jitc-2025-011682 (PMC12142029; doi:10.1136/jitc-2025-011682)
Supplement: online supplemental file 1 [file jitc-13-5-s001.docx]

**Supplementary File**

**Willsmore et al.** *Circulating immunoregulatory B cell and autoreactive antibody profiles predict lack of toxicity to anti-PD-1 checkpoint inhibitor treatment in advanced melanoma*

**Supplementary Figures**

**Supplementary Figure 1**


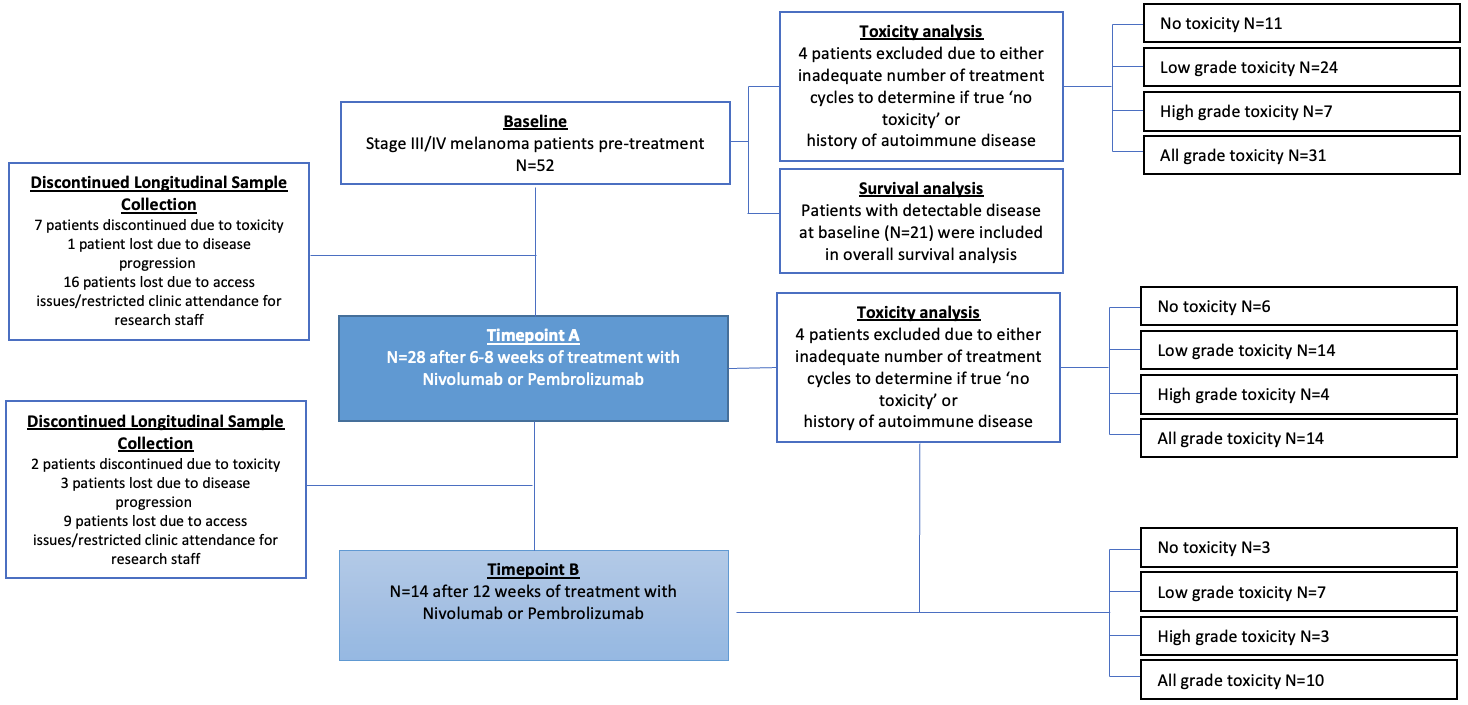

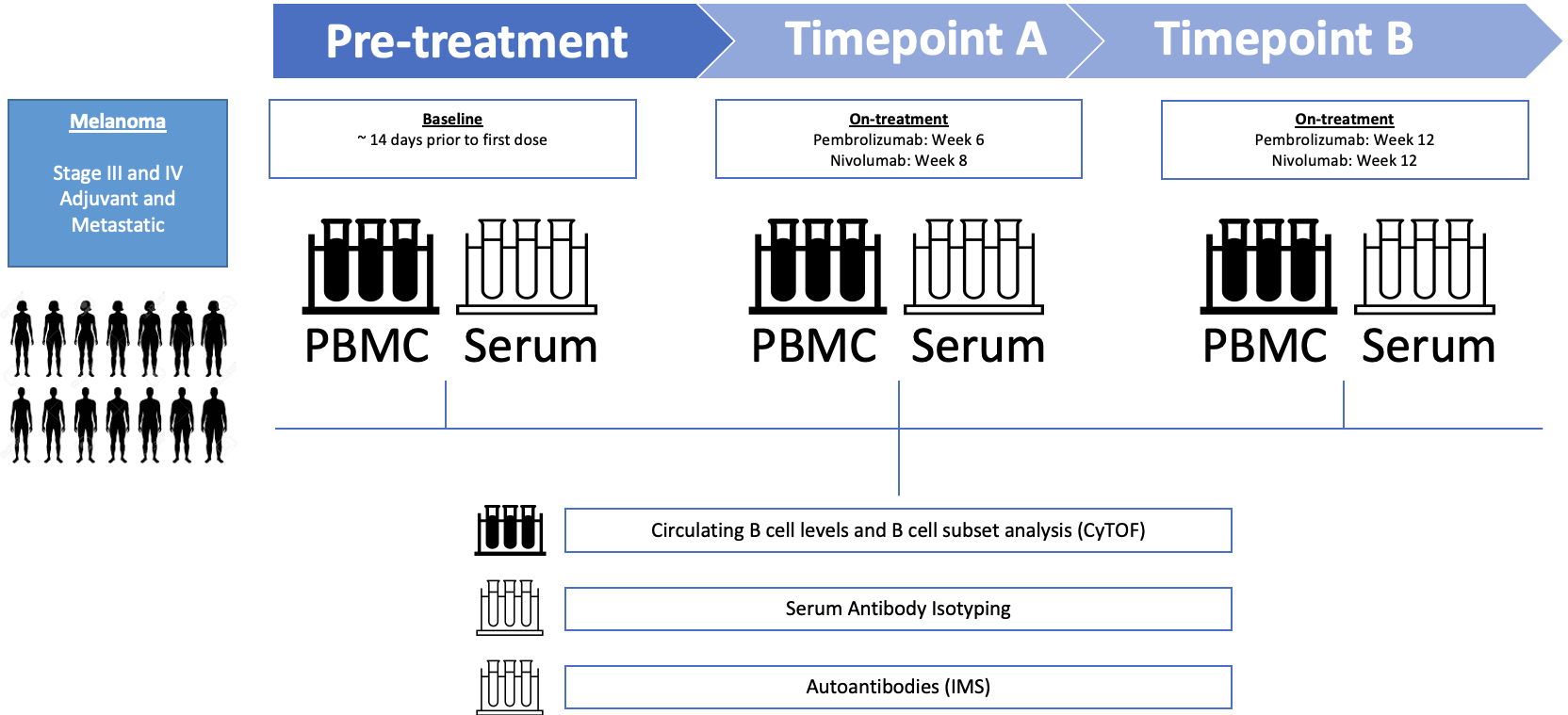


**A**

**B**

**Supplementary Figure 1: Flowcharts illustrating the melanoma patient cohort studies for baseline and for longitudinal analysis with mass cytometry.** (**A**) Patient peripheral blood sampling protocol. Patients with stage III/IV melanoma planned to receive checkpoint inhibitor therapy were recruited and baseline peripheral blood sampling was performed. Subsequent on-treatment sampling was performed according to anti-PD-1 treatment protocol: Pembrolizumab at week 6 and week 12; Nivolumab at week 8 and week 12. (**B**) Experimental flowchart detailing the patient samples collected at baseline and longitudinally, and numbers of patients included at different sampling timepoints, and description of attrition (numbers lost to discontinued treatment, disease progression and access to clinics, including during the COVID-19 pandemic, as well as evaluations undertaken to study B cell signatures in the acquired samples. PBMC: peripheral blood mononuclear cells. CyTOF: Cytometry by time of flight; IMS: Immuno-Mass Spectrometry.

**Supplementary Figure 2**


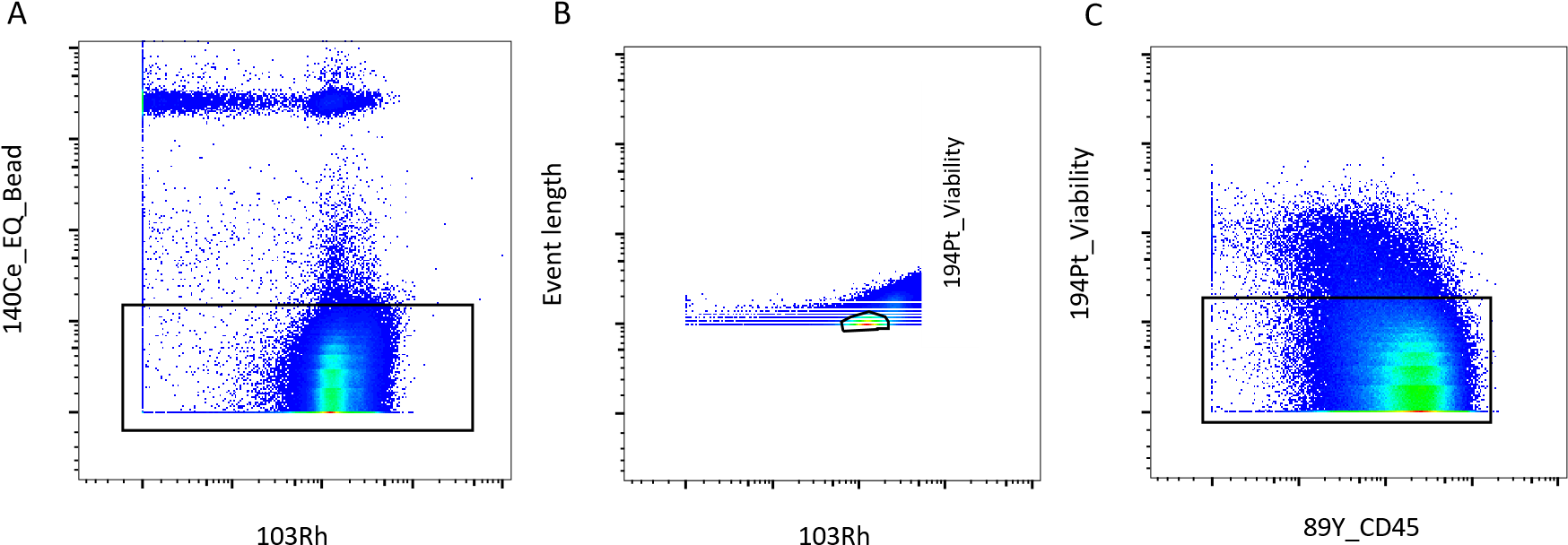

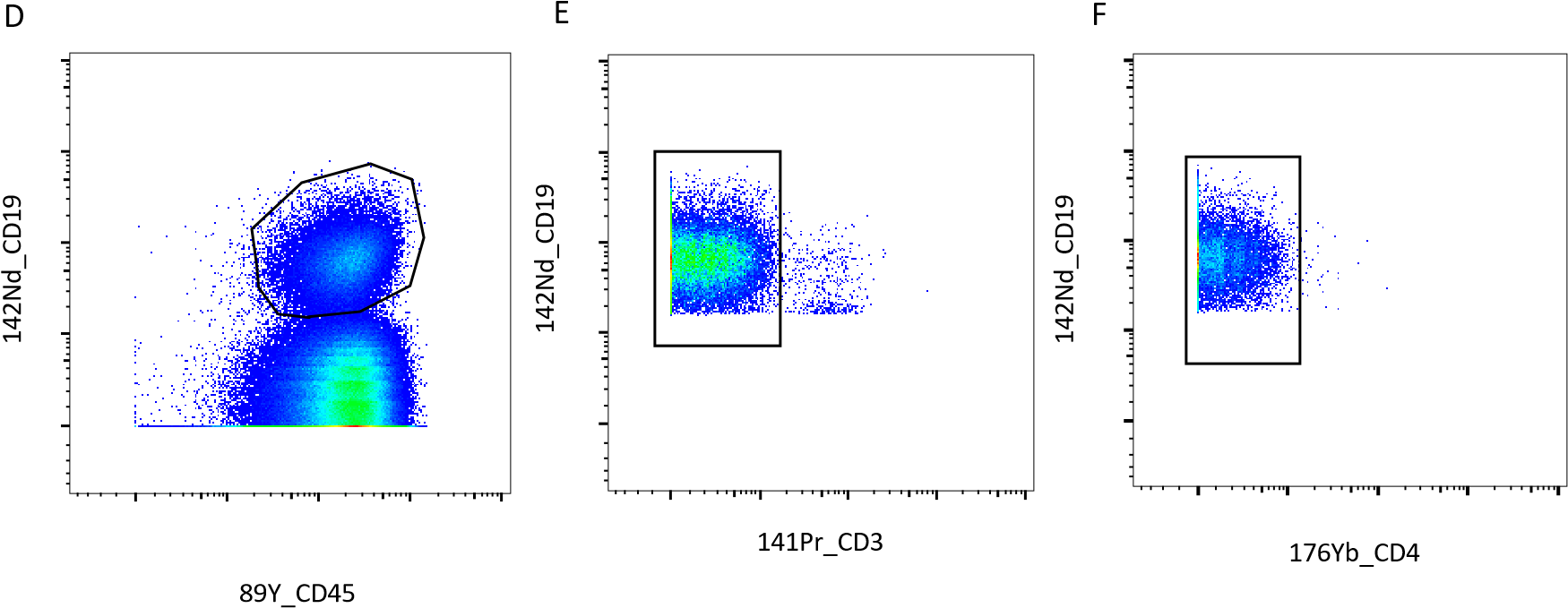

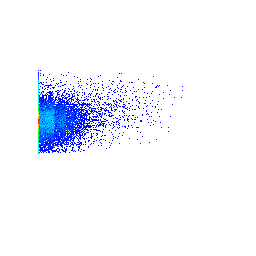


142Nd_CD19

209Bi_CD16

**Supplementary Figure 2: Gating strategy to identify B cells for CyTOF mass cytometry analyses.** Ungated events were sequentially gated in FlowJo software to identify the live singlet population from PBMCs. The B cell population was identified, 140Ce_EQ_Beads (polystyrene bead standards) were removed. 194PT live/dead stain and CD45 were used to identify live cells. Event length and 103Rh DNA were used to identify singlets, CD19 and CD45 were used to identify live singlet B cells. Dot plots of the CD19 and CD3 markers were used to exclude T cells. Dot plots of CD19 and CD4 markers were used to exclude T cells and CD16 was used to exclude monocytes.

**Supplementary Figure 3**

**Transitional**

**Naïve CD21lo**

**Class Switched**

**Memory IgG+**

**Double Negative**

**(CD27-IgD-)**

**IL-10+ Plasmablast**

**Naïve PD-L1+ TGFB+**

**A**

**B**

**Supplementary Figure 3: Peripheral blood B cells evaluated by mass cytometry and merged into canonical populations exhibit regulatory features in advanced melanoma patient versus healthy volunteer blood.** (**A**) Comparison of the relative abundance of CD19+ cells out of the total CD45+ population, in HV, and in melanoma patients stratified by stage of disease, at baseline prior to CPI treatment. (**B**) Comparison of the relative abundance of B cell populations identified by mass cytometry and merged into 10 canonical clusters, Figure 1), in HV and melanoma patients stratified by stage of disease confirmed enriched plasmablast, naïve, DN and immunosuppressive B cell populations and reduced class-switched memory phenotypes in advanced melanoma. HV: Healthy volunteers; CPI: checkpoint inhibitor (anti-PD-1); DN: double negative. *p<0.05; **p<0.01.

**Supplementary Figure 4**

**B**

**Transitional**

**IL-10+ Plasmablast**

**Double Negative (CD27-IgD-)**

**No Toxicity**

**High Grade Toxicity**

**C**

**A**

**D**

**Supplementary Figure 4: Baseline B cell phenotypes predict immune related anti-PD-1 related adverse events.** (**A**) Comparison of the relative abundance of CD19+ cells out of the total CD45+ population, in melanoma patients stratified by toxicity status. (**B**) B cell clusters identified by mass cytometry (Figure 2) were merged into 10 clusters, identifying patterns of differences between pre-treatment B cell profiles of patients who develop ‘no toxicity’ versus those with ‘high grade toxicity’ to anti-PD-1 therapy. Merged B cell populations are visualised in a bar chart showing the relative proportion of merged clusters per sample. (**C**) Relative abundance of merged B cell clusters that are statistically significantly differentially expressed between ‘no toxicity’ and ‘high grade toxicity’ groups at baseline. *p<0.05; **p<0.01. (**D**) Kaplan-Meir curve demonstrating no significant association between development of toxicity and overall survival in melanoma patients with metastatic disease (stratified overall survival according to the presence of ‘high grade toxicity’ (n=5, ESMO grading 3) and ‘no toxicity’ (n=8) p=0.3049.

**Supplementary Figure 5**


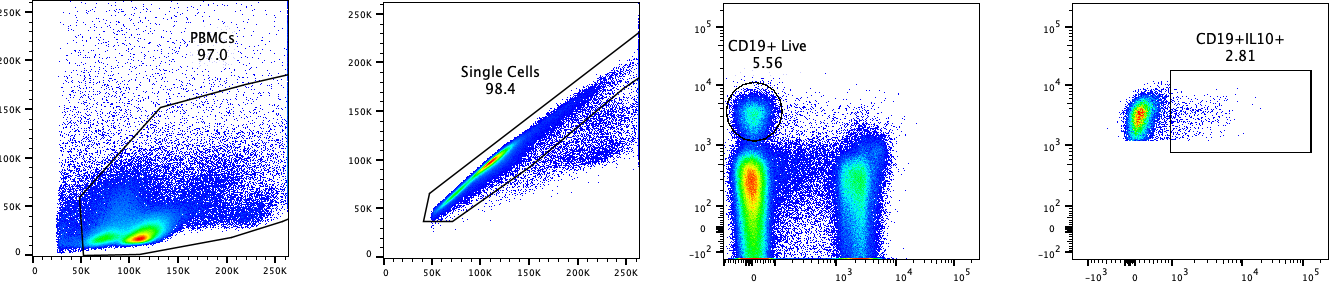


SSc

FSc-H

CD19 APC

CD19 APC

FSc

FSc-A

Live/Dead

IL-10 PE

**C**

**B**

**Baseline**

Blood collected from patients with stage III/IV disease pre-treatment n=20

**Timepoint B: Clinical observation follow up**

12 weeks of treatment with Pembrolizumab, Nivolumab, Avelumab or Ipilimumab/Nivolumab

N=14

**Toxicity analysis**

High toxicity n=7

Low toxicity n=7

6 patients excluded due to missing baseline information/data

PBMC analysis for CD19+IL-10+ B cells

**A**

**Supplementary Figure 5. Lower levels of IL-10^+^ B cells at baseline are detected in individuals who experienced high grade toxicity to subsequent treatment with checkpoint immunotherapy.** (**A**) Flowchart illustrating the PAIR cancer patient cohort at baseline and at 12 weeks post treatment. High and low toxicity were defined based on an internal multiparametric grading tool, using the affected organs, the severity of toxicities and the total toxicity burden. (**B**) Gating strategy to detect CD19+IL-10+ B cells in blood. (**C**) Frequency of IL-10 producing B cells at baseline timepoint in patients who developed high- and low-grade toxicity after checkpoint inhibition treatment. PBMCs were treated with CpG (1 mM) + IL-2 (25 U/ml) overnight and after 3-hour stimulation with PMA (phorbol myristate acetate) plus Ionomycin, IL-10 intracellular staining was performed. * p<0.05.

**Supplementary Figure 6**

**Supplementary Figure 6:** Heatmap representing putative serum autoantibody target proteins, generated by Immuno-mass spectrometry (IMS) of serum immunoglobulins (IgG) from melanoma patients prior to anti-PD-1 treatment, stratified into two cohorts: those that develop any grade toxicity versus those who do not develop toxicity. Heatmap signal intensity corresponds to IMS peak area for each protein.

**Supplementary Figure 7**

**Supplementary Figure 7: Paired comparison of the relative abundance of CD19+ cells out of the total CD45+ population, in melanoma across baseline and treatment timepoints, stratified by stage of disease.** Statistical analysis using a two-way ANOVA. * p<0.05.

**Supplementary Figure 8**

High B cell fold change after 1 cycle of anti-PD-1 therapy

Low B cell fold change after 1 cycle of anti-PD-1 therapy

**A**

**B**

**Supplementary Figure 8: A decline in circulating B cells on treatment with anti-PD-1 therapy was associated with earlier onset of adverse events and poorer overall survival.** (**A**) Correlation between changes in circulating B cells and days to onset of toxicity in patients receiving anti-PD-1 therapy. Statistical correlation determined by linear regression analysis. B cell decline associated with earlier onset of toxicity. *p<0.05. (**B**) Kaplan-Meier plot showing the probability of survival in patients with either high or low circulating B cell fold change after 1 cycle of anti-PD-1 therapy. Cut-off points for high versus low fold change were statistically determined after generation of receiver-operator curves (ROC), cut-off points for high vs low fold change were 0.87. B cell fold change of >0.87 after 1 cycle of treatment was associated with improved survival *p<0.05.

**Supplementary Figure 9**

**
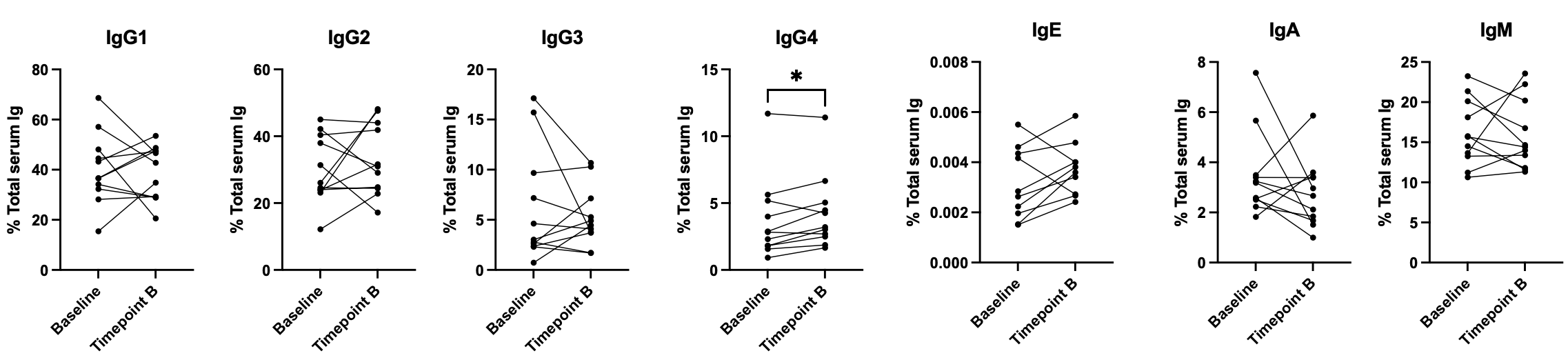
**

**Supplementary Figure 9: Paired statistical comparison of circulating antibody isotypes performed comparing fold change on treatment; and antibody isotype as percentage of total circulating antibody titre at timepoint B on treatment.** Statistical analysis using the Wilcoxon paired rank test. *p<0.05, **p<0.01, ***p<0.001.

**Supplementary Figure 10**


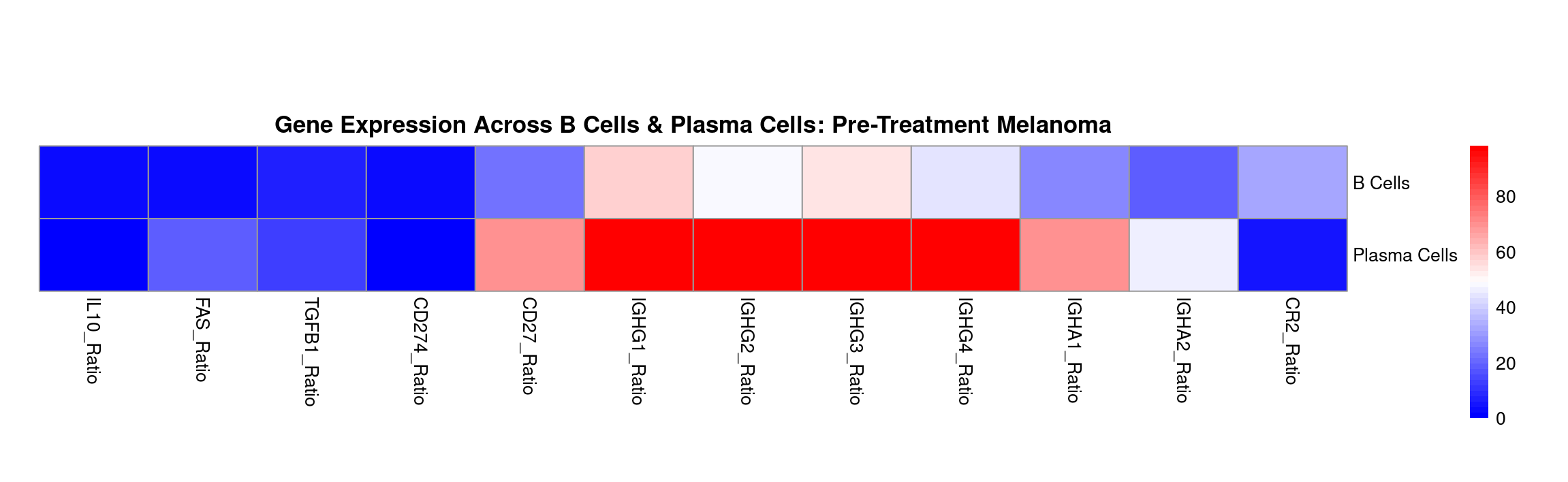

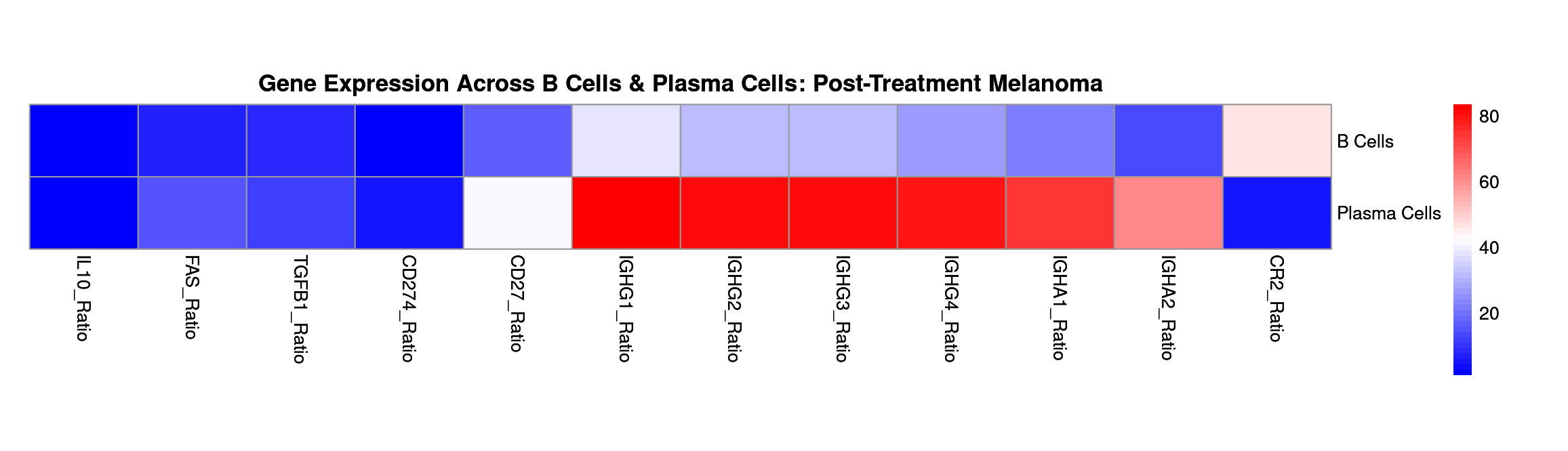


**A**

**B**


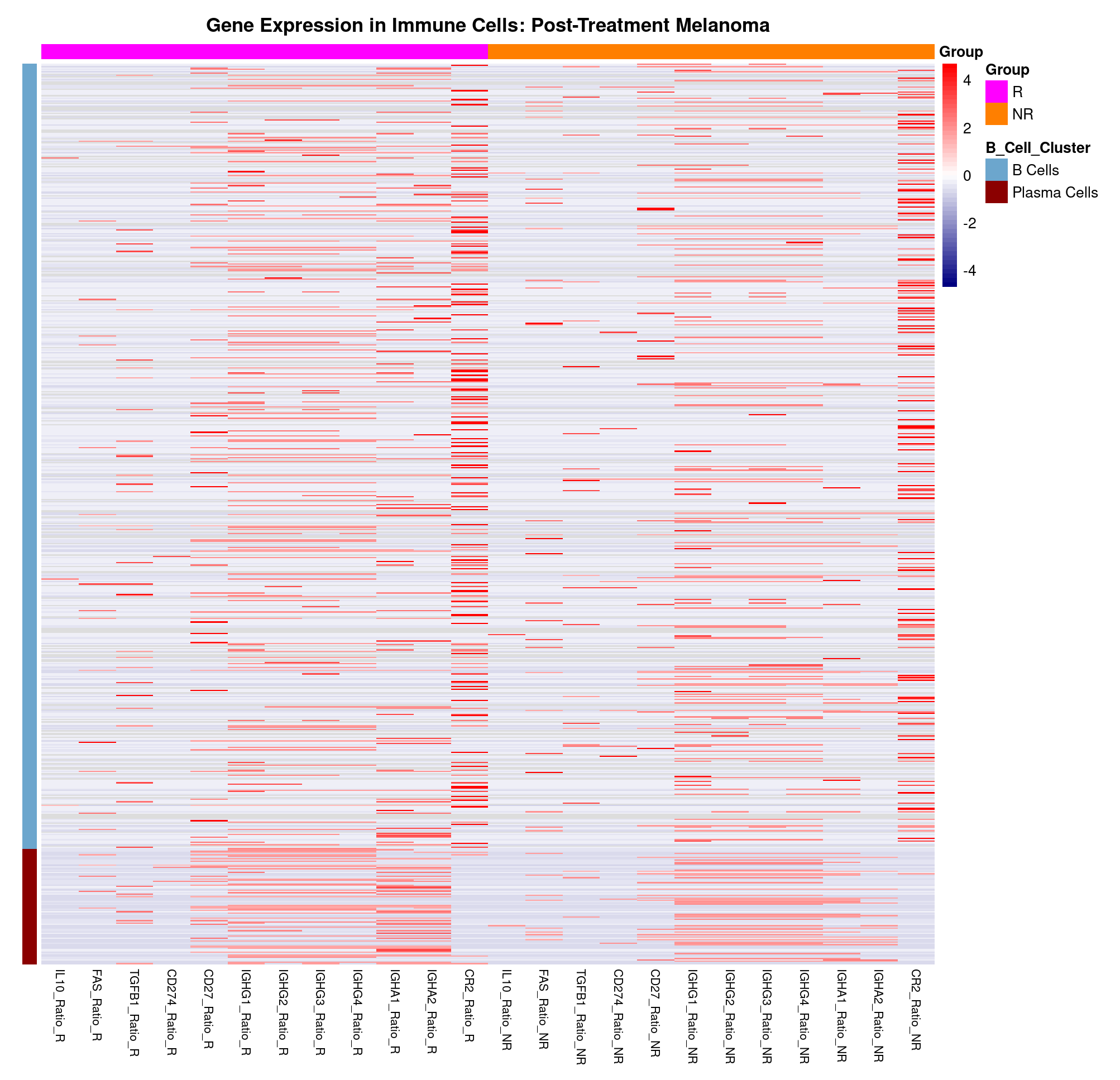

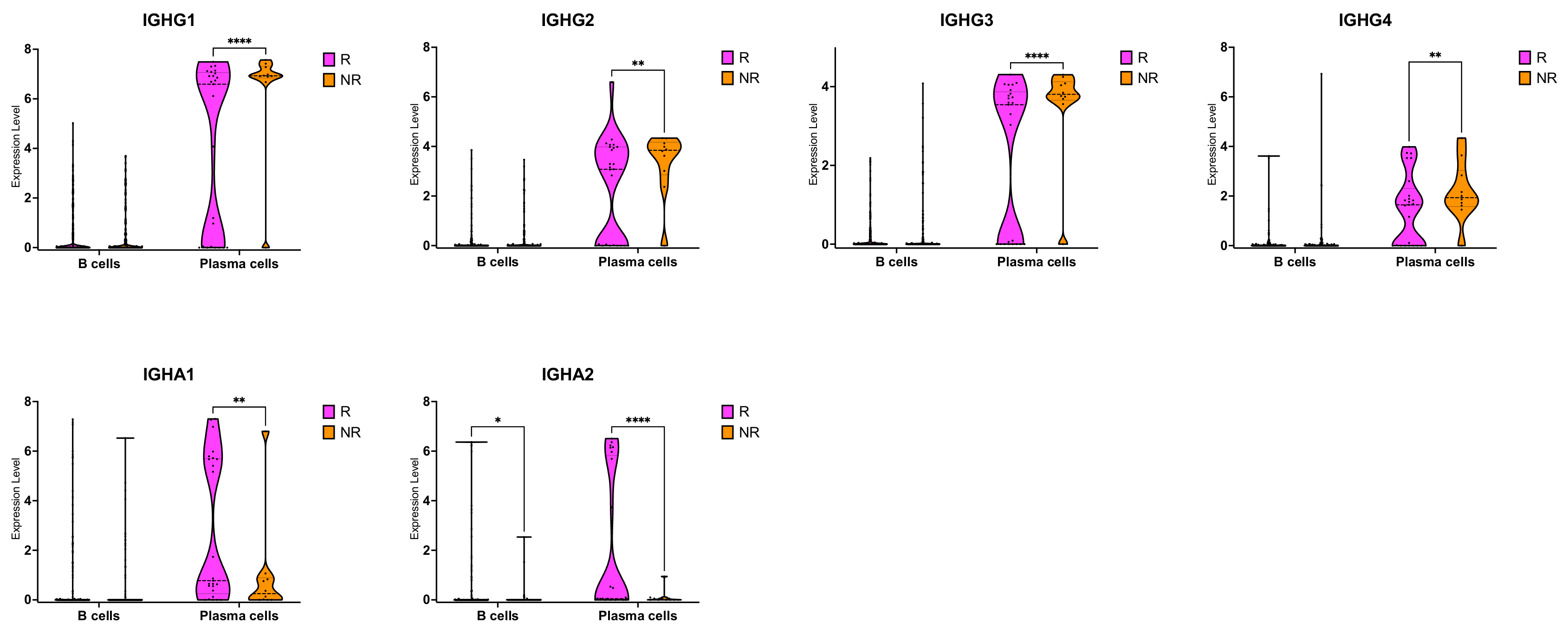

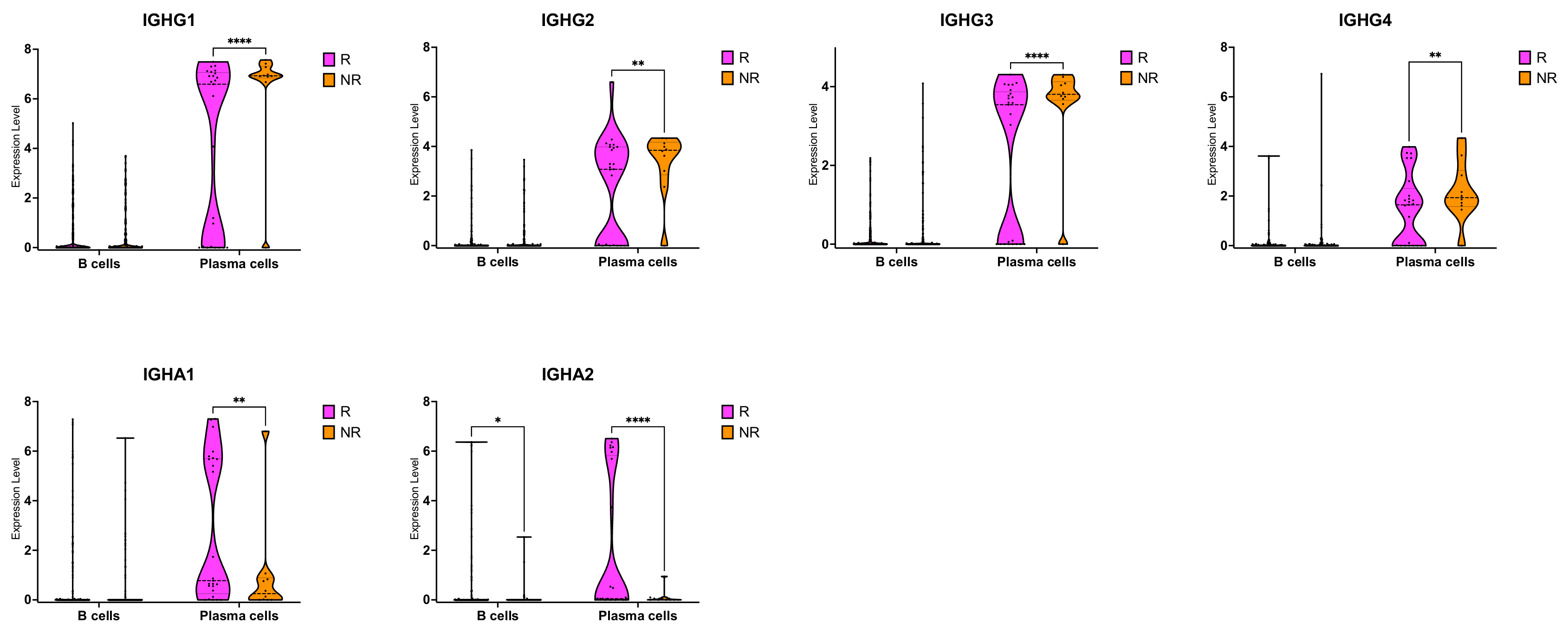


**E**

**F**


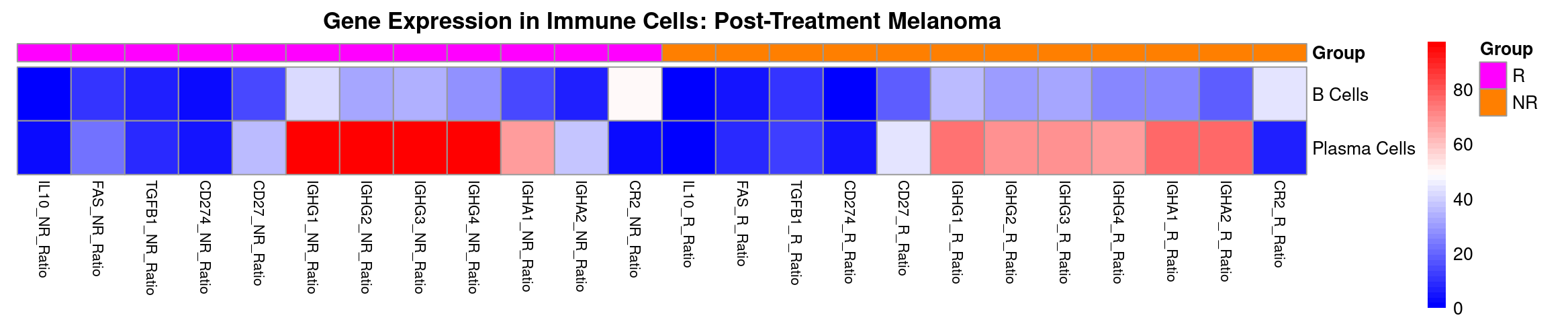

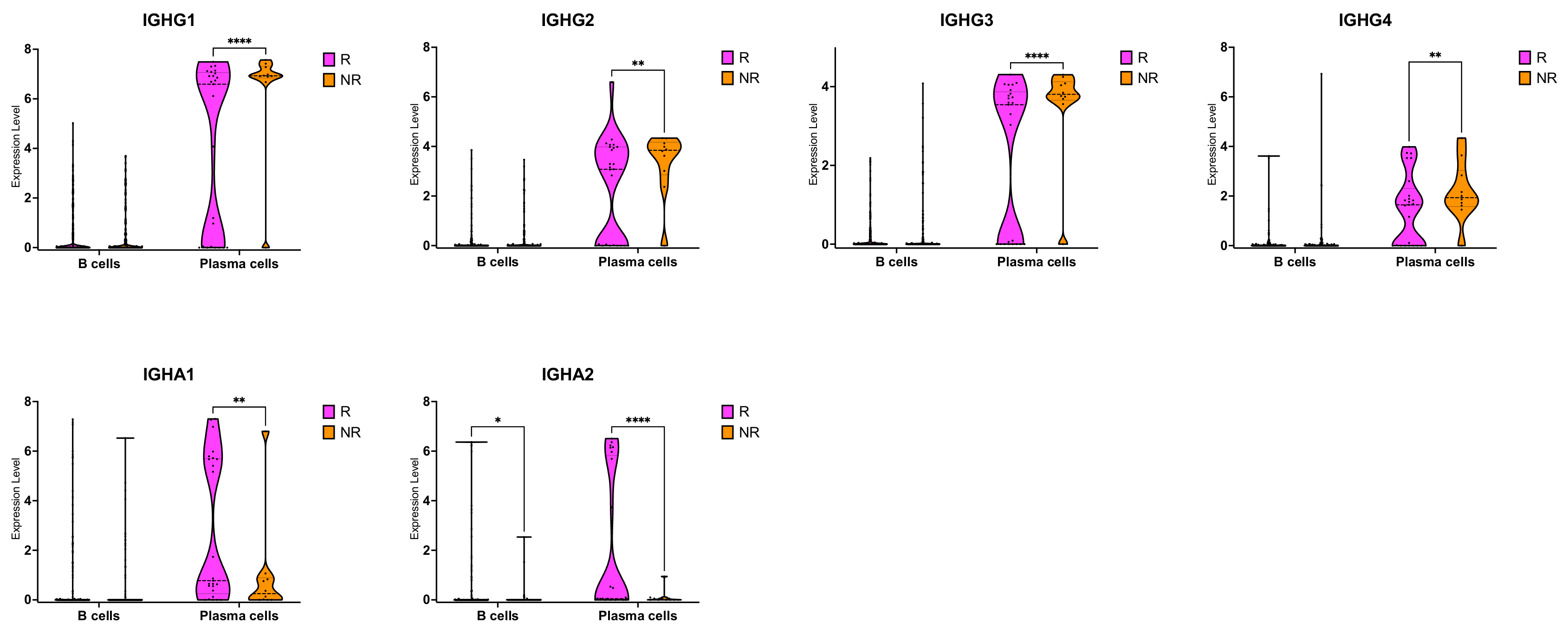


**G**

**C**

**D**

**
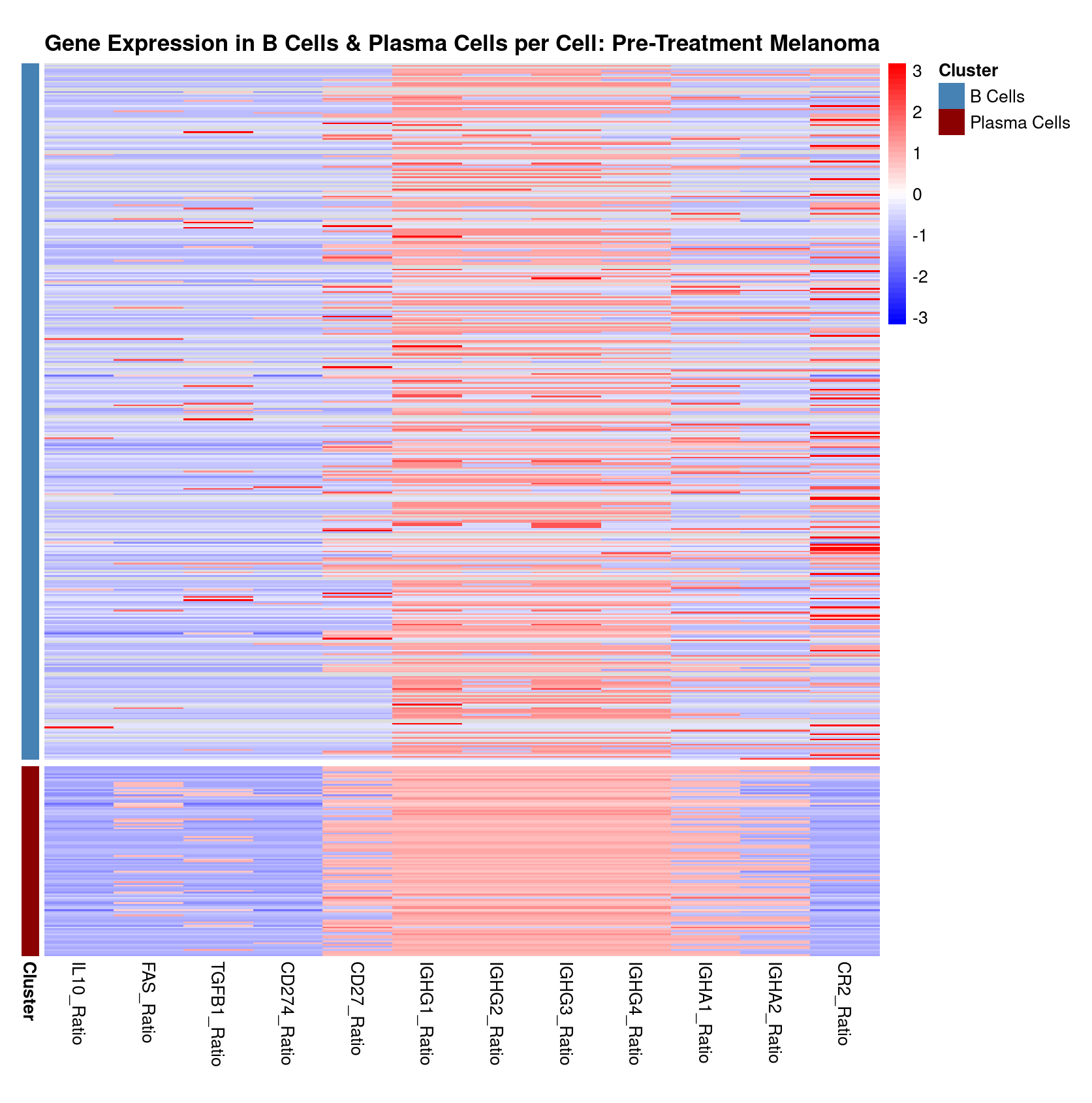

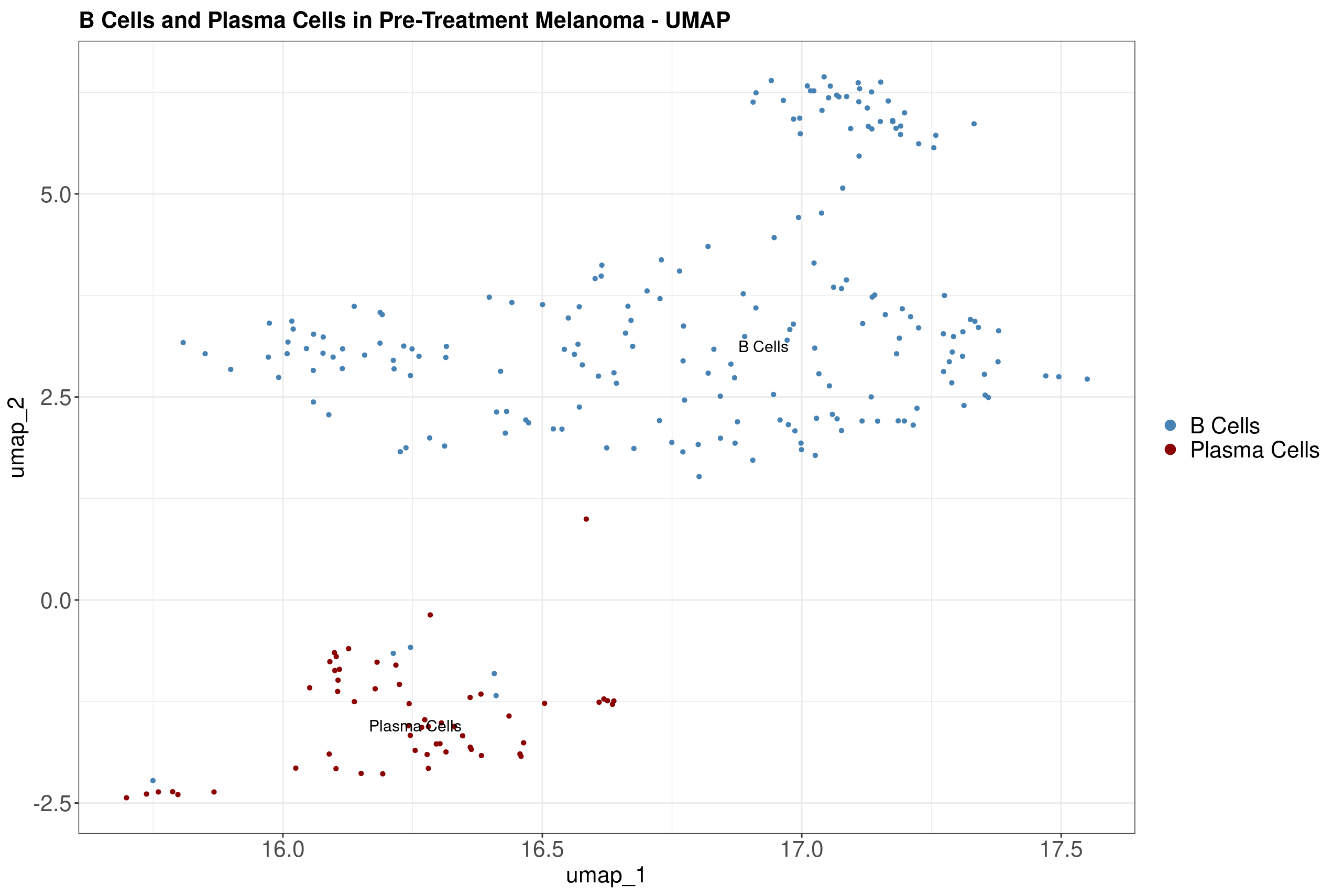

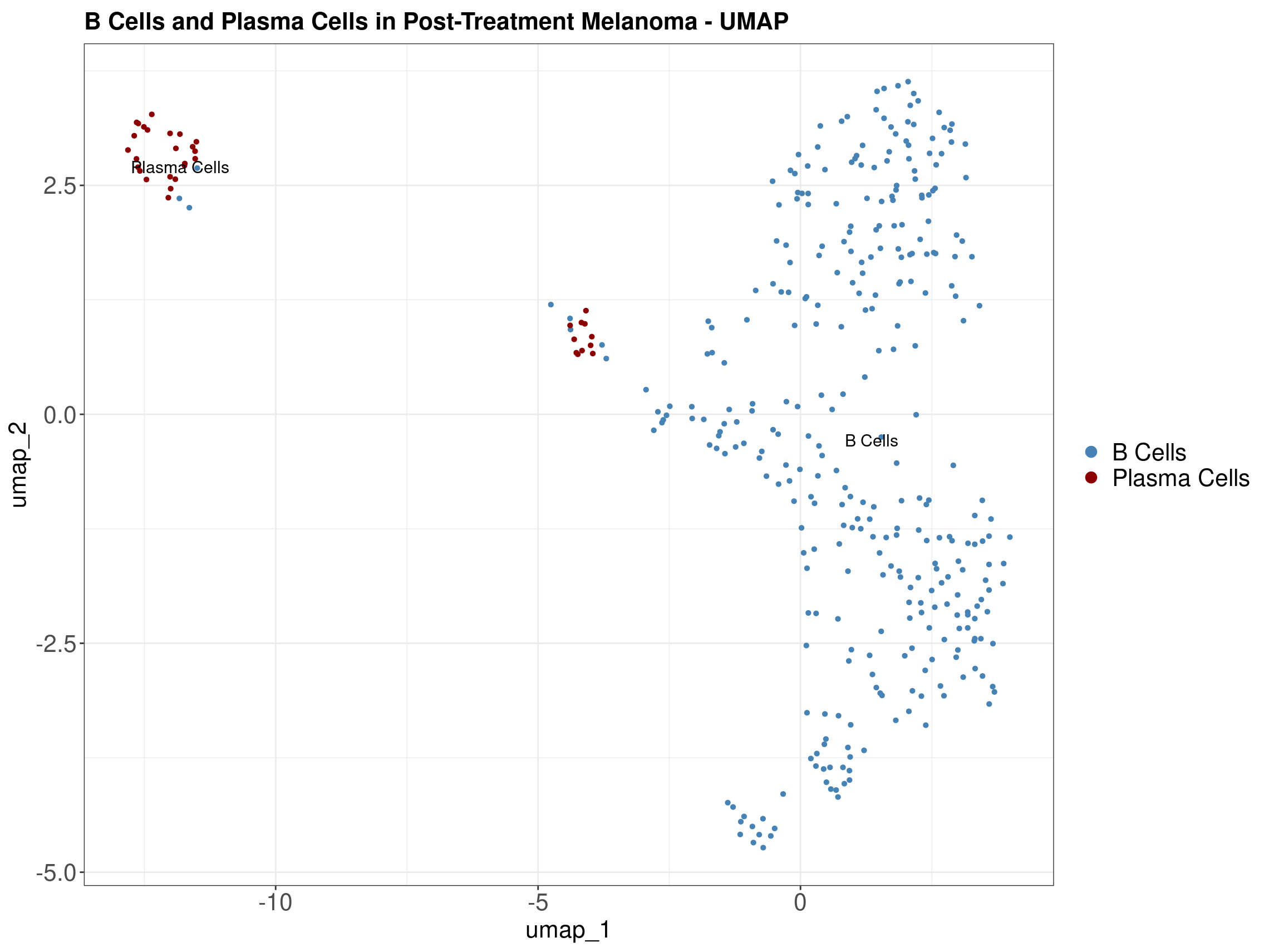

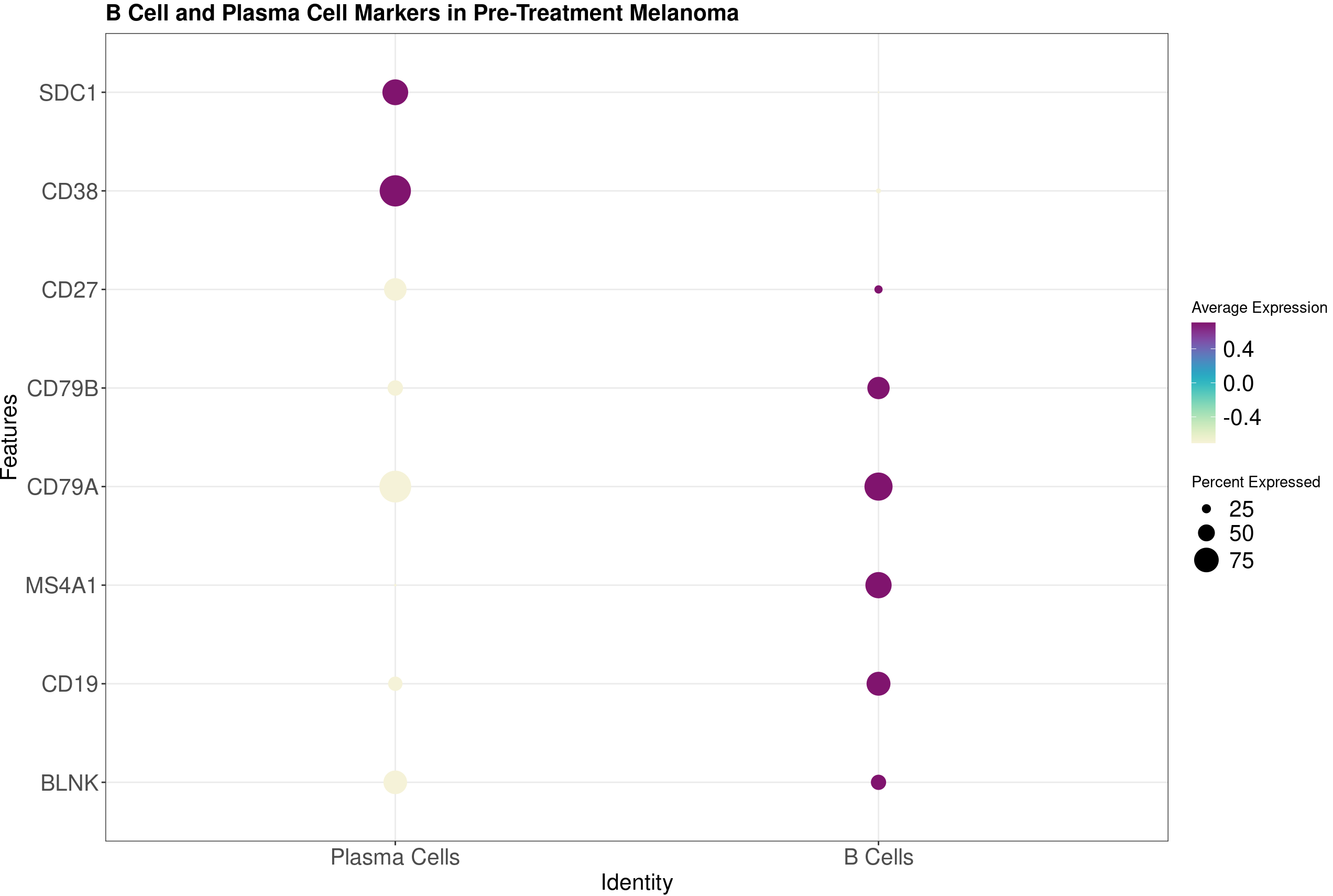

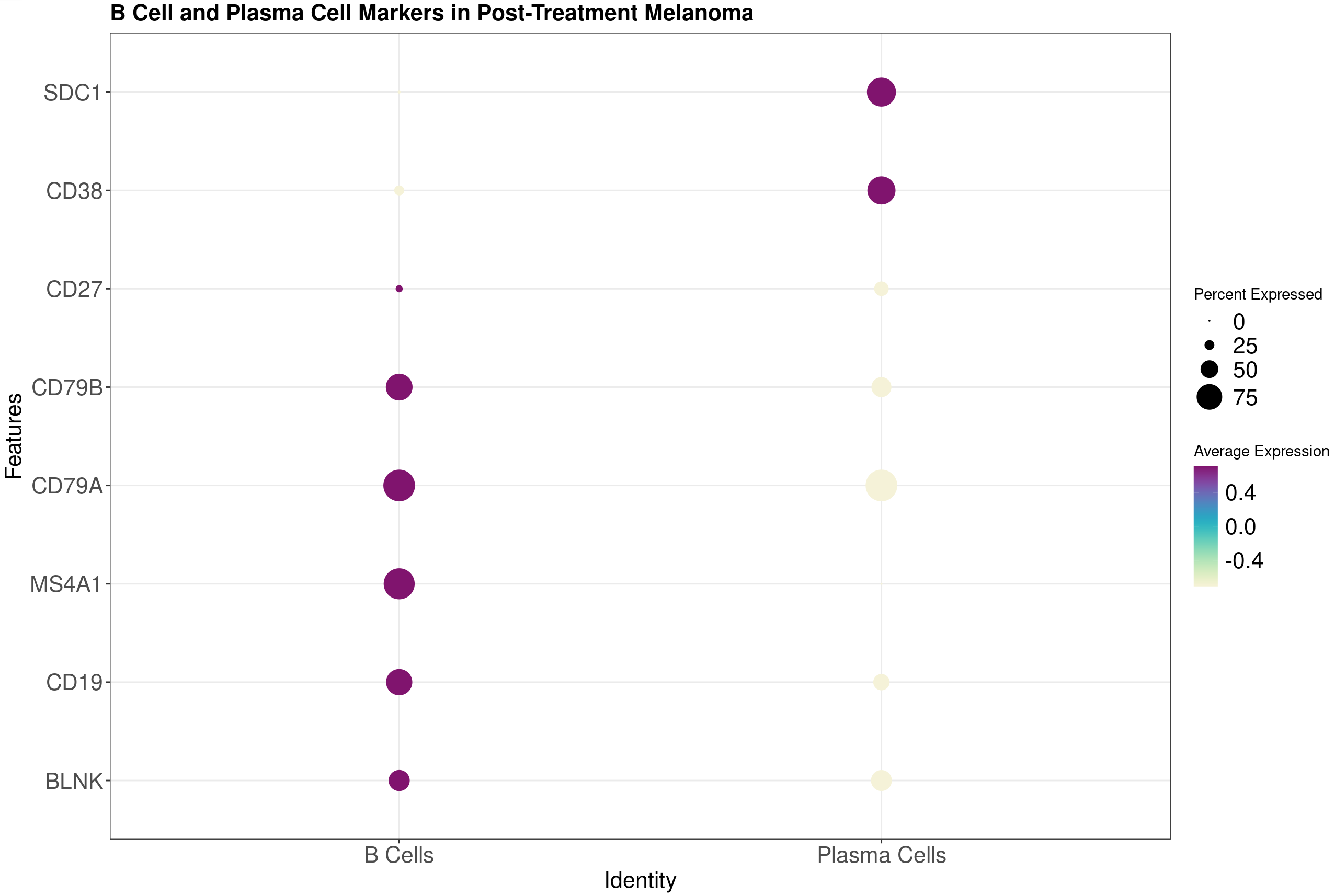
**

**
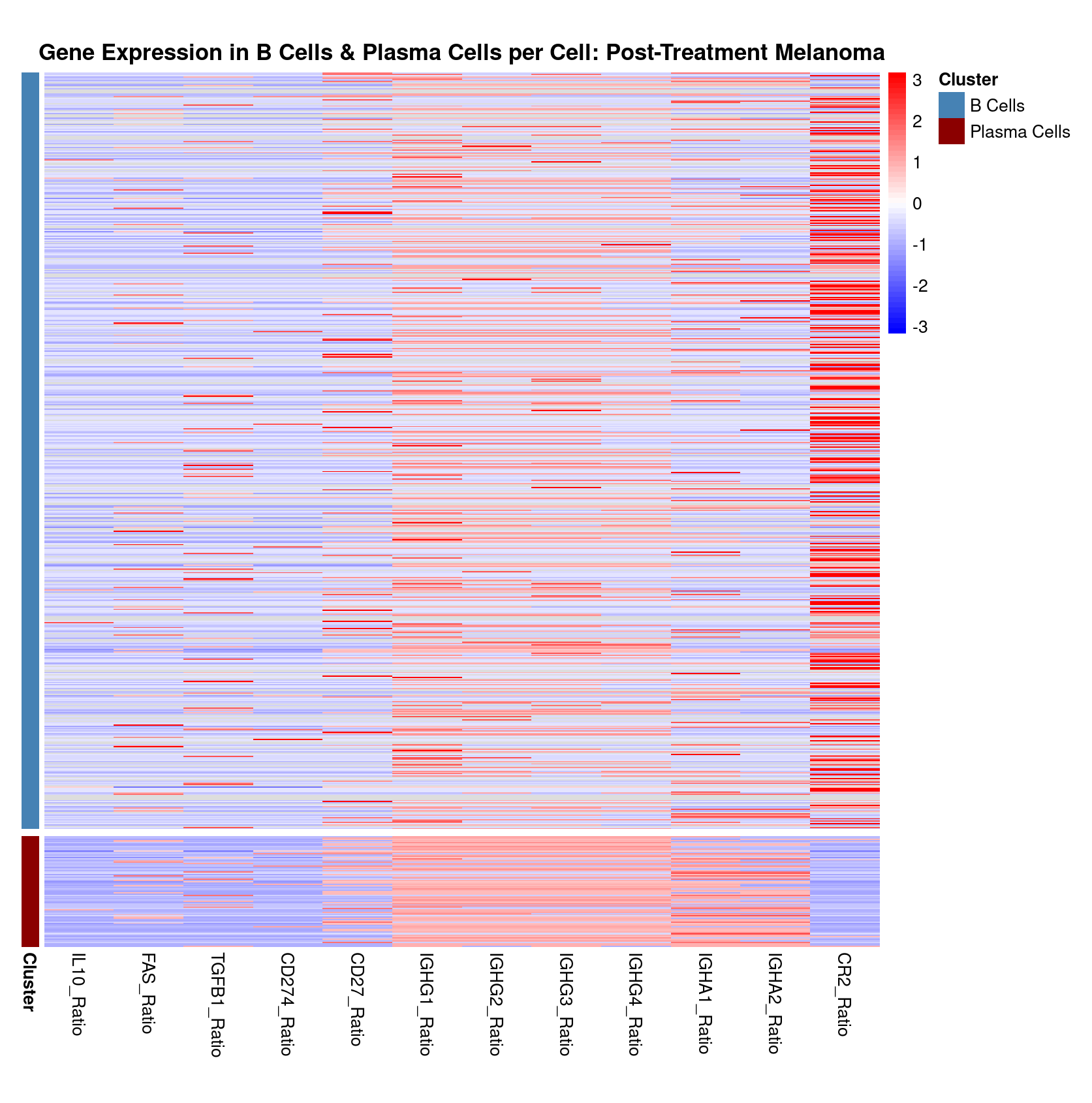
**

**Supplementary Figure 10: scRNA-Seq analysis of melanoma samples shows expression of markers across plasma cell and B cell clusters at pre-treatment and post-treatment and post-treatment stratified by Responders and Non-Responders to checkpoint inhibitor immunotherapy.** (**A**) Melanoma samples pre-treatment: Left: UMAPs showing cell type annotation in melanoma samples; Right: Dot plots indicating cell type markers in each cluster. (**B**) Melanoma samples post-treatment: Left: UMAP of B cell and plasma cell type annotation in post-treatment melanoma cells; Right: Dot plots indicating cell type markers in each cluster. (**C-D**) Heatmaps show ratio of positive gene expression across plasma cell and B cell clusters (C), and per cell (D), in pre- and post-treatment melanoma samples. (**E-F**) In samples post-treatment with checkpoint inhibitor immunotherapy, from patients who were categorised as Responders (R) and Non-Responders (NR) post-treatment: Heatmaps show ratio of positive gene expression across total plasma cell and B cell clusters (E) and per individual cell (F). (**G**) Violin plots show expression of IGHG and IGHA isotype genes in B cell and plasma cell clusters in R and NR patients, post-treatment. Statistical analysis performed using two-way ANOVA. * P ≤ 0.05; ** P ≤ 0.01; *** P ≤ 0.001; **** P ≤ 0.0001.

**Supplementary Tables**

**Supplementary Table 1**: Summary of melanoma patient characteristics and sampling and treatment details. Patients in this cohort did not receive neoadjuvant therapy. NK: not known; BCC: basal cell carcinoma; COPD: chronic obstructive pulmonary disease; T2DM: type 2 diabetes mellitus.

| **Patient ID** | **AJCC Stage** | **Relevant previous medical history** | **Previous systemic treatment for melanoma** | **Resected (RD) vs Active disease (AD) at baseline** | **Checkpoint inhibitor** | **Baseline sample collection timepoint (days pre-treatment)** |
| --- | --- | --- | --- | --- | --- | --- |
| M1 | IIIB | Nil | Nil | RD | Pembrolizumab | 29 |
| M2 | IIIC | Melanoma (second primary), papillary thyroid cancer, ulcerative colitis | Nil | AD | Pembrolizumab | 19 |
| M3 | IIIC | Emphysema, hypertension, ischaemic heart disease | Nil | AD | Pembrolizumab | 14 |
| M4 | IV | Hypertension, prostatic hypertrophy and hypercholesterolemia | Nil | AD | Pembrolizumab | 13 |
| M5 | IV | Nil | Nil | RD | Pembrolizumab | 26 |
| M6 | IV | BCCs, hypertension, hyperlipidaemia, thoracic sympathectomy, ischaemic heart disease | Nil | AD | Pembrolizumab | 0 |
| M7 | IV | Ischaemic heart disease, hypercholesterolaemia, T2DM | Nil | AD | Pembrolizumab | 19 |
| M8 | IV | Nil | Nil | AD | Pembrolizumab | 18 |
| M9 | IV | Hypercholesterolaemia | Dabrafenib and Trametinib | AD | Pembrolizumab | 11 |
| M10 | IIIB | Nil | Nil | AD | Nivolumab | 19 |
| M11 | IV | COPD | Nil | AD | Pembrolizumab | 12 |
| M12 | IIIC | Osteoarthritis, polio | Dabrafenib | AD | Pembrolizumab | 11 |
| M13 | IIID | Hypothyroidism, hypercholesterolaemia | Nil | AD | Pembrolizumab | 19 |
| M14 | IV | Nil | Nil | AD | Pembrolizumab | 22 |
| M15 | IV | Hypertension | Nil | AD | Pembrolizumab | 378 |
| M16 | IV | Previous melanoma, hypertension, osteoarthritis | Nil | AD | Nivolumab | 14 |
| M17 | IIIC | Breast cancer | Nil | RD | Nivolumab | 53 |
| M18 | IIIB | Gaucher’s disease | Nil | RD | Pembrolizumab | 33 |
| M19 | IIIA | Nil | Nil | RD | Nivolumab | 34 |
| M20 | IIID | Atrial fibrillation, transient ischaemic attach, ischaemic heart disease | Nil | RD | Pembrolizumab | 0 |
| M21 | IV | Breast cancer | Nil | RD | Nivolumab | 32 |
| M22 | III | Nil | Nil | RD | Pembrolizumab | 4 |
| M23 | IIIC | Hypothyroidism, hypercholesterolaemia | Dabrafenib and Trametinib | RD | Pembrolizumab | 18 |
| M24 | IIIC | Hypertension | Nil | RD | Pembrolizumab | 18 |
| M25 | IIIA | Depression, hypercholesterolaemia | Nil | RD | Nivolumab | 14 |
| M26 | IV | Prostate cancer, hypertension | Nil | AD | Pembrolizumab | 15 |
| M27 | IV | T2DM, hypertension, heart failure | Nil | AD | Pembrolizumab | 13 |
| M28 | IIIB | Nil | Nil | RD | Pembrolizumab | 26 |
| M29 | III | Melanoma (second primary), Parkinson’s disease | Nil | RD | Pembrolizumab | 15 |
| M30 | IV | T2DM, hypertension, hyperlipidaemia | Nil | AD | Pembrolizumab | 14 |
| M31 | IIIC | T2DM, hypercholesterolaemia, | Nil | RD | Pembrolizumab | 14 |
| M32 | IV | BCC, Bowen’s disease | Nil | AD | Pembrolizumab | 12 |
| M33 | IIIC | Hypertension, hyperlipidaemia, arrhythmia, cataracts | Nil | AD | Pembrolizumab | 19 |
| M34 | IV | Nil | Nil | AD | Pembrolizumab | 12 |
| M35 | IIID | Nil | Dabrafenib and Trametinib | AD | Pembrolizumab | 7 |
| M36 | IV | Prostate cancer, hypertension | Nil | AD | Pembrolizumab | 12 |
| M37 | IV | Ulcerative colitis | Nil | AD | Pembrolizumab | 0 |
| M38 | IV | Ischaemic heart disease, hypertension, hypercholesterolaemia | Nil | AD | Pembrolizumab | 12 |
| M39 | IV | T2DM | Nil | AD | Pembrolizumab | 13 |
| M40 | IIIC | Nil | Nil | AD | Pembrolizumab | 12 |
| M41 | IIIC | Gout, hypertension | Nil | RD | Pembrolizumab | 14 |
| M42 | IIIC | Ischaemic heart disease, hypertension, hypercholesterolaemia, T2DM | Nil | NK | Pembrolizumab | 5 |
| M43 | IV | Osteoarthritis, rheumatoid arthritis,vitiligo, hypothyroidism | Nil | AD | Pembrolizumab | 25 |
| M49 | III | Nil | Nil | RD | Pembrolizumab | 2 |
| M45 | IIIC | Breast cancer, Parkinson’s disease, osteoporosis | Nil | AD | Pembrolizumab | 14 |
| M46 | IV | Nil | Vemurafenib | AD | Pembrolizumab | 5 |
| M47 | IIIC | Nil | Nil | AD | Pembrolizumab | 6 |
| M48 | IV | Ulcerative colitis | Nil | AD | Nivolumab | 47 |
| M49 | IV | Hypertension, hyperlipidaemia | Nil | AD | Nivolumab | 14 |
| M50 | IV | Osteoarthritis | Nil | AD | Pembrolizumab | 0 |
| M51 | III | Ischaemic heart disease, transitional cell carcinoma | Nil | RD | Pembrolizumab | 12 |
| M52 | IIIC | Hypertension, hyperlipidaemia | Nil | RD | Pembrolizumab | 19 |

**Supplementary Table 2:** Summary of patient characteristics in the high-grade toxicity cohort (n=18) followed up for the onset of toxicity to anti-PD-1 checkpoint immunotherapy. Median time to severe toxicity was 93 days (range 36-182 days). No patients received concomitant immunosuppressants. NA: not applicable. G: grade of toxicity as per Common Terminology for Adverse Events Criteria; T2DM: type 2 diabetes mellitus.

| **Patient ID** | **Stage** | **Relevant previous medical history** | **Previous systemic treatment for melanoma** | **Toxicity status** | **Previous history of autoimmune disease** | **Nature of toxicity (Grade of toxicity)** |
| --- | --- | --- | --- | --- | --- | --- |
| MT1 | IV | Ischaemic heart disease, hypercholesterolaemia, T2DM | Nil | No Toxicity | Nil | NA |
| MT2 | IV | Hypercholesterolaemia | Dabrafenib and Trametinib | No Toxicity | Nil | NA |
| MT3 | IIID | Hypothyroidism, hypercholesterolaemia | Nil | Toxicity | Nil | Arthralgia (G3) |
| MT4 | IV | Hypertension | Nil | Toxicity | Nil | Myositis (G3), diarrhoea (G1), fatigue (G1) |
| MT5 | IV | Breast cancer | Nil | No Toxicity | Nil | NA |
| MT6 | IIIC | Hypertension | Nil | Toxicity | Nil | Mucositis (G3), hypophysitis (G2), hypothyroidism (G2) |
| MT7 | IV | T2DM, hypertension, heart failure | Nil | No Toxicity | Nil | NA |
| MT8 | IIIC | Hypertension, hyperlipidaemia, arrhythmia, cataracts | Nil | No Toxicity | Nil | NA |
| MT9 | IV |  | Nil | No Toxicity | Nil | NA |
| MT10 | IIIC | Breast cancer, Parkinson’s disease, osteoporosis | Nil | No Toxicity | Nil | NA |
| MT11 | IV | Hypertension, hyperlipidaemia | Nil | No Toxicity | Nil | NA |
| MT12 | IV | Osteoarthritis | Nil | Toxicity | Nil | Diarrhoea (G3) |
| MT13 | III | Ischaemic heart disease, transitional cell carcinoma | Nil | Toxicity | Nil | Diarrhoea (G3) |
| MT14 | IIIC | Hypertension, hyperlipidaemia |  | No Toxicity | Nil | NA |
| MT15 | IIIC | Nil | Nil | No Toxicity | Nil | NA |
| MT16 | IIIB | Nil | Nil | Toxicity | Nil | Myositis/polymyalgia rheumatic (G3), pneumonitis (G2) |
| MT17 | IIIC | Hypothyroidism, hypercholesterolaemia | Dabrafenib and Trametinib | Toxicity | Nil | Myositis (G3), arthralgia (G2) |
| MT18 | IV | Nil | Nil | No Toxicity | Nil | NA |

**Supplementary Table 3**: CyTOF panel markers used for phenotyping of gated CD19+ cells.

| **Metal label** | **Antigen** |
| --- | --- |
| Nd144 | CD38 |
| Nd145 | CD81 |
| Nd146 | IgD |
| Sm147 | CD20 |
| Sm149 | CD25 |
| Nd150 | CD138 |
| Eu151 | HLA-DR |
| Sm152 | CD21 |
| Sm154 | IgG |
| Gd155 | PD-1 |
| Gd156 | PD-L1 |
| Gd158 | CD10 |
| Tb159 | CD22 |
| Dy161 | CD5 |
| Dy162 | CD79B |
| Dy163 | BCL-6 |
| Dy164 | CD95 |
| Ho165 | CD40 |
| Er166 | IL-10 |
| Er167 | CD27 |
| Er168 | Ki-67 |
| Tm169 | CD24 |
| Er170 | TGF-Beta (TGFβ) |
| Yb171 | CXCR5 |
| Yb172 | IgM |

**Supplementary Table 4:** Summary of patient serum samples used for immuno-mass spectrometry analysis.

| **Timepoint** | **Pembrolizumab** | **Nivolumab** | **Total number of samples** |
| --- | --- | --- | --- |
| Baseline | 23 | 7 | 30 |
| Timepoint A | 16 | 5 | 21 |
| Timepoint B | 9 | 4 | 13 |

**Supplementary Table 5.** Summary of patient characteristics in an additional cohort (PAIR) followed up for the onset of toxicity to checkpoint immunotherapy. Patients with advanced solid tumours treated with checkpoint inhibitor immunotherapy. RCC: renal cell carcinoma, NSCLC: non-small cell lung cancer, SqCLC: squamous cell lung cancer, TCC: transitional cell carcinoma. NA: not available.

| **Patient ID** | **Tumour** | **Stage** | **Relevant previous medical history** | **Previous systemic anti-cancer treatment** | **Resected disease (RD) vs. Active disease (AD) at baseline** | **Subsequent checkpoint inhibitor** |
| --- | --- | --- | --- | --- | --- | --- |
| PAIR1 | Melanoma | IV | NA | NA | AD | Nivolumab |
| PAIR2 | RCC | IV | NA | NA | AD | Ipilimumab/Nivolumab |
| PAIR3 | RCC | IV | NA | NA | AD | Ipilimumab/Nivolumab |
| PAIR4 | NSCLC | IV | Rheumatoid arthritis | NA | AD | Pembrolizumab |
| PAIR5 | Melanoma | IV | NA | NA | AD | Ipilimumab/Nivolumab |
| PAIR6 | Melanoma | IV | Rheumatoid arthritis | NA | AD | Nivolumab |
| PAIR7 | Merkel cell carcinoma | IV | NA | NA | AD | Avelumab |
| PAIR8 | Melanoma | IV | NA | Dabrafenib/Trametinib | AD | Ipilimumab/Nivolumab |
| PAIR9 | SqCLC | IV | NA | Gemcitabine/Carboplatin | AD | Pembrolizumab |
| PAIR10 | RCC | IV | NA | Pazopanib | AD | Nivolumab |
| PAIR11 | Melanoma | IV | NA | NA | AD | Pembrolizumab |
| PAIR12 | Mesothelioma | IIIB | NA | Cisplatin/Pemetrexed | AD | Pembrolizumab |
| PAIR13 | RCC | IV | NA | Pazopanib, Cabozantinib | AD | Nivolumab |
| PAIR14 | Melanoma | IV | NA | NA | AD | Ipilimumab/Nivolumab |
| PAIR15 | NSCLC | IV | NA | Cisplatin/Pemetrexed | AD | Atezolizumab |
| PAIR16 | RCC | IV | NA | NA | AD | Ipilimumab/Nivolumab |
| PAIR17 | RCC | IV | NA | Sunitinib | AD | Nivolumab |
| PAIR18 | NSCLC | IV | Crohn’s disease | NA | AD | Pembrolizumab |
| PAIR19 | RCC | IV | NA | NA | AD | Ipilimumab/Nivolumab |
| PAIR20 | TCC | IV | NA | Gemcitabine/Cisplatin, Cabozantinib (clinical trial) | AD | Pembrolizumab |

**Supplementary Table 6:** Univariate and pairwise-adjusted logistic regression for B cell clusters in patient circulation, adjusting individually for age, sex, or stage.

| **Term** | **OR** | **CI_lower** | **CI_upper** | **P_value** | **adj** |
| --- | --- | --- | --- | --- | --- |
| **cluster_4** | 0.250664354921864 | 0.039551055992891 | 0.883180768311118 | 0.0773587509875699 | univariate |
| **cluster_4** | 0.232085192548841 | 0.0334192500395524 | 0.866505114027299 | 0.0753313147789673 | age |
| **cluster_4** | 0.259789971999987 | 0.041139214036948 | 0.885055387870285 | 0.0820315790625773 | sex |
| **cluster_4** | 0.283897180710908 | 0.0428292007748341 | 1.11411848216581 | 0.124828236244398 | stage_clean |
| **cluster_7** | 0.0715804177140457 | 0.00274976060778253 | 0.514642313562088 | 0.0412960840953451 | univariate |
| **cluster_7** | 0.0758823516792653 | 0.00274145264753503 | 0.511930881025293 | 0.0409755203912915 | age |
| **cluster_7** | 0.0710277150806406 | 0.00276569313502282 | 0.520071681681924 | 0.0396408924192496 | sex |
| **cluster_7** | 0.00534410217282653 | 6.80165972304803E-08 | 0.231224620171072 | 0.10767567885572 | stage_clean |
| **cluster_12** | 0.0536928825372736 | 0.000852012982894872 | 0.668864675573936 | 0.0877431518383623 | univariate |
| **cluster_12** | 0.0526477788857509 | 0.000867691508828579 | 0.637946760267712 | 0.0809973721487329 | age |
| **cluster_12** | 0.0333507363402261 | 0.000176599423350919 | 0.599011911734111 | 0.0921823360922484 | sex |
| **cluster_12** | 0.0406286175282927 | 0.000317462129384672 | 0.573311107871404 | 0.0854217185971267 | stage_clean |
| **cluster_10** | 0.0254008619067452 | 0.000213419480875979 | 0.524602631019609 | 0.0612681659289993 | univariate |
| **cluster_10** | 0.0249109512231746 | 0.000193089715609904 | 0.522524609707182 | 0.0618457227453359 | age |
| **cluster_10** | 0.0191002776062106 | 0.000102184143551074 | 0.476756928732377 | 0.0591739033079833 | sex |
| **cluster_10** | 0.0119831652064435 | 5.13136754199252E-05 | 0.40730441692277 | 0.0436164891505863 | stage_clean |
| **cluster_9** | 0.299820668212271 | 0.0579866443914671 | 0.898822654381915 | 0.0682674496655672 | univariate |
| **cluster_9** | 0.297993202888505 | 0.055251994453332 | 0.898148246120004 | 0.0702052087074633 | age |
| **cluster_9** | 0.230301228631913 | 0.033789656626545 | 0.778412292653847 | 0.0515143723871196 | sex |
| **cluster_9** | 0.320494114762799 | 0.0656574834082791 | 1.00120425940983 | 0.0850762334515303 | stage_clean |
| **cluster_6** | 2.81956789913832E-07 | 3.02137548894894E-16 | 0.0120536987111625 | 0.0441623411436824 | univariate |
| **cluster_6** | 3.26367439290233E-07 | 8.58927714197327E-16 | 0.0100890854933025 | 0.0376315227679604 | age |
| **cluster_6** | 1.84110285824208E-108 | 0 | 5.22806368048123E-37 | 0.336884528490379 | sex |
| **cluster_6** | 5.10121312715705E-09 | 7.16119493394566E-25 | 0.00539196802410946 | 0.0835349833885651 | stage_clean |
| **cluster_11** | 0.513557015436982 | 0.228824271036963 | 0.907461529960339 | 0.049409997063175 | univariate |
| **cluster_11** | 0.482574179168635 | 0.193707529470792 | 0.885226525098675 | 0.047985228513558 | age |
| **cluster_11** | 0.509793226643716 | 0.214295958096451 | 0.916753049037154 | 0.0569477707930915 | sex |
| **cluster_11** | 0.518064189916621 | 0.214868944013921 | 0.960197857010929 | 0.0708207607728561 | stage_clean |
| **cluster_5** | 4.31829565333235 | 1.44283438054954 | 26.9531635834394 | 0.0395975211177322 | univariate |
| **cluster_5** | 5.13283024082316 | 1.51659544630067 | 41.04255936334 | 0.0419448085942499 | age |
| **cluster_5** | 4.42824700977125 | 1.47220382372797 | 31.8875660386712 | 0.0423495418708748 | sex |
| **cluster_5** | 5.95555793564932 | 1.57093067055256 | 66.6399648672928 | 0.0429580251491841 | stage_clean |
| **cluster_23** | 7.30783699578145 | 1.61376684499477 | 72.6937828245551 | 0.0341625256289093 | univariate |
| **cluster_23** | 9.29846405351373 | 1.80917730673619 | 130.786986675207 | 0.0330011679950519 | age |
| **cluster_23** | 7.28998505127082 | 1.58916995316216 | 73.2022178557717 | 0.0349542121998092 | sex |
| **cluster_23** | 7.07968630862552 | 1.42217764440863 | 84.0924259379376 | 0.0477325595359419 | stage_clean |

**Supplementary Table 7:** Univariate and pairwise-adjusted logistic regression for antibody isotype, adjusting individually for age, sex, or stage.

| **Antibody isotype** | **OR** | **CI_lower** | **CI_upper** | **P_value** | **adj** |
| --- | --- | --- | --- | --- | --- |
| **IgG1** | 1.053942 | 0.972957 | 1.169355 | 0.243431 | univariate |
| **IgG1** | 1.051861 | 0.965241 | 1.176098 | 0.294839 | age |
| **IgG1** | 1.075513 | 0.984066 | 1.240022 | 0.179236 | sex |
| **IgG1** | 1.039978 | 0.961521 | 1.15132 | 0.364545 | stage_clean |
| **IgG2** | 0.986183 | 0.908015 | 1.069611 | 0.721069 | univariate |
| **IgG2** | 0.98904 | 0.910554 | 1.07811 | 0.782231 | age |
| **IgG2** | 0.968948 | 0.885003 | 1.056403 | 0.454101 | sex |
| **IgG2** | 1.001177 | 0.921692 | 1.089593 | 0.976415 | stage_clean |
| **IgG3** | 1.093768 | 0.868811 | 1.519092 | 0.496632 | univariate |
| **IgG3** | 1.121191 | 0.878703 | 1.602176 | 0.427591 | age |
| **IgG3** | 1.09759 | 0.851613 | 1.603201 | 0.539166 | sex |
| **IgG3** | 0.992759 | 0.715902 | 1.427475 | 0.96413 | stage_clean |
| **IgG4** | 0.575531 | 0.268635 | 0.90349 | 0.064334 | univariate |
| **IgG4** | 0.572556 | 0.27057 | 0.905678 | 0.054898 | age |
| **IgG4** | 0.514422 | 0.189774 | 0.915065 | 0.093763 | sex |
| **IgG4** | 0.579475 | 0.253445 | 0.932073 | 0.087747 | stage_clean |
| **IgE** | 0 | 0 | 1.29488786549152e-46 | 0.05575 | univariate |
| **IgE** | 0 | 0 | 1.08089738921704e-56 | 0.057139 | age |
| **IgE** | 0 | 0 | 1.39415723814118e-282 | 0.101106 | sex |
| **IgE** | 6.72530157969573e-300 | 0 | 7195576805134726144 | 0.11082 | stage_clean |
| **IgA** | 0.705939 | 0.312637 | 1.364076 | 0.317029 | univariate |
| **IgA** | 0.643113 | 0.210416 | 1.667381 | 0.378455 | age |
| **IgA** | 0.695514 | 0.263841 | 1.414016 | 0.368308 | sex |
| **IgA** | 0.663767 | 0.259979 | 1.333445 | 0.297899 | stage_clean |
| **IgM** | 0.91938 | 0.752587 | 1.088754 | 0.348185 | univariate |
| **IgM** | 0.916678 | 0.752549 | 1.087936 | 0.332238 | age |
| **IgM** | 0.910074 | 0.732348 | 1.095186 | 0.332815 | sex |
| **IgM** | 0.935798 | 0.764436 | 1.111219 | 0.462918 | stage_clean |

**Supplementary Materials and Methods**

**Serum collection and processing**

Whole blood samples were collected (10ml volume per patient or HV subject) in 10ml yellow-capped serum separating (SST) collection tubes and allowed to clot for a minimum of 30 minutes. The tubes were then spun at 3000rpm (1500xg) for 10 minutes at 4 degrees Celsius (°C) and the serum layers were immediately transferred in 200µl aliquots to the -80°C freezer in cryovials for storage. Vials were removed and thawed on ice as required for experimental work.

**Peripheral blood mononuclear cell isolation and cryopreservation**

Peripheral blood mononuclear cell isolation and cryopreservation was performed in line with a validated laboratory protocol as previously published [1-4]. Whole blood samples were collected (15-50ml per patient or HV subject) in 10ml purple-capped ethylenediaminetetraacetic acid (EDTA)-coated tubes and kept on a roller-shaker prior to further processing, for a maximum of 4 hours. The blood was then diluted at a 1:1 ratio in phosphate buffered saline (PBS) in 50ml Falcon™ tubes and subsequently layered over Ficoll-Paque^TM^ PLUS before spinning at 1040g for 20 minutes at room temperature without a brake. The peripheral blood mononuclear cell (PBMC) layer was then harvested using Pasteur pipettes and washed in PBS at 1020g for 10 minutes. Cells were subsequently washed with Red Cell Lysis Buffer at 370g for 5 minutes. After discarding the supernatant and resuspending the pellet, cells were washed again with 10ml RPMI at 370g for 5 minutes. Isolated PBMCs were aliquoted with freezing Solution A and B (FBS (Foetal Bovine Serum), RPMI1640, P/S (Penicillin/Streptomycin, 10,000 U/mL)) and DMSO (Dimethyl sulfoxide), at 4:4:1 ratio) and stored in 1ml aliquots at 10x10^6^ cells/ml at -80°C at -1°C/minute. Samples were transferred to liquid nitrogen for cryogenic storage until the time of assaying.

**Thawing of PBMCs for mass cytometry analyses**

Cryovials of frozen PBMCs were removed and thawed in a water bath at 37°C when required for staining. When only a few ice crystals remained visible, cells were aspirated using a sterile Pasteur pipette and added to 10ml warmed RMPI1640 cell culture medium supplemented with 20% FBS. Cells were washed with 50ml of RPMI1640 at 300g for 10 minutes. Cells were incubated for 1 hour at 37°C with Benzonase (Merck 70664-3) at 1µl for every 1x10^6^ cells and subsequently washed again with RPMI as above. PBMC cell counts were performed using a haemocytometer with trypan blue (1:10 dilution in PBS).

**Mass cytometry cell staining and data acquisition**

After thawing, cells were counted with the aim of staining 2-4 x 10^6^ cells per tube, where cell yield was higher the samples were split into 2 aliquots. Thawed PBMCs were added to filter-cap FACS tubes (Fisher Scientific) and washed with 3ml of MaxPar Cell Staining Buffer (Fluidigm) at 800g for 5 minutes. Cell pellets were re-suspended in 80µl buffer, added to a FACS tube and cells were incubated with 5µl of Fc-Blocking Solution (Human Trustain Fc blocking solution (Biolegend)) for 10 minutes at room temperature. All antibodies were vortexed and spun at 10,000g for 5 minutes prior to adding to each cell sample to prevent possible antibody aggregates. Antibodies for cell surface staining were added to the cells and cells were vortexed gently before incubating for 30 minutes at room temperature. Cisplatin at 5µM used as a viability stain was added for the last minute of the surface staining incubation. The cells were quenched with 4ml of MaxPar cell staining buffer (Fluidigm) and washed at 800g for 5 minutes. The cells were then prepared for intracellular staining. The cells were fixed using 1ml of MaxPar Fix I buffer (Fluidigm) at room temperature for 15 minutes. The fixed cells were washed twice in MaxPar Perm S Permeabilisation buffer (Fluidigm). Next, the intracellular antibody staining cocktail was prepared and added to the cells for 30 minutes at room temperature. After washing with MaxPar cell staining buffer, cells were incubated overnight at 4°C with intercalation solution consisting of DNA intercalator 103-Ir (Fluidigm) and MaxPar Fix and Perm Buffer (Fluidigm). Finally, after overnight incubation cells were washed with MaxPar Cell staining Buffer (Fluidigm) and twice in Milli-Q water at 800g for 5 minutes immediately prior to acquisition on a Helios™ Mass Cytometer (Fluidigm). Samples were resuspended in Milli-Q water with polystyrene bead standards at a concentration of 0.5 x10^6^ cells/ml. All samples were run on a Helios™ Mass Cytometer. Data was acquired at a rate of 300 – 400 events per second, with a target of 500,000 – 750,000 events per sample. Each batch of samples included a healthy control along with patient samples for quality control.

**Mass cytometry and cluster analysis using the FlowSOM clustering algorithm**

Data were obtained in the form of .fcs files from the Helios™ Mass Cytometer. Files were normalised and concatenated using Fluidigm CyTOF Software. Pre-processed files were uploaded to FlowJo software for gating. EQ beads, 103Rh (DNA intercalator) and event length parameter were used to determine intact DNA+ singlets from debris and cell aggregates.^194^ Cisplatin live/dead stain was then used to identify live intact singlets.

B cell populations were determined by gating on CD45+ CD19+ populations and then refined further for doublet exclusion using the markers: CD45, CD19, CD8a, CD4, CD3 and CD16a, to determine a ‘True’ CD19+ population (Supplementary Figure 2). After identifying the CD19+ population of interest using FlowJo software, the CD19+ .fcs were uploaded in R for downstream analyses using modified script from the CATALYST, diffCYT, FlowSOM, edgeR  and flowCORE packages which can be found using the Bioconductor terminal <https://www.bioconductor.org/packages/release/bioc/vignettes/CATALYST/inst/doc/differential.html>. B cell phenotypes were determined using an unsupervised FlowSOM clustering algorithm that utilises a Self-Organising Map to evaluate marker expression on cells in a high-dimensional space, therefore generating well segregated B cell subsets [5]. From samples pooled together for clustering, distinct metaclusters were defined according to median scaled expression, as previously described [6].

**Transcriptomic analyses of B cell and plasma cell markers in melanoma specimens**

Publicly available scRNA-Seq data were downloaded from GEO under the accession code GSE120575 [7]. The data was split into pre-treatment (n=12) and post-treatment (n=19) patient cohorts, with treatment including either anti-PD1 or a combination of anti-PD1 and anti-CTLA4 antibodies. A Seurat object was created using the CreateSeuratObject() function and log normalized using NormalizeData with a scale factor = 10000. Data was scaled and centred. Principal component analysis was performed using 2000 variable genes identified by function FindVariableFeatures (method = “vst”). The first 15 principal components were selected for downstream analysis. A resolution of 1 was used to determine clusters using the FindClusters() function. Clusters identified as B cells and plasma cells were used for subsequent analyses. Dot plots using key B and plasma cell markers were plotted using the Dotplot() function. Violin plots were generated using the VlnPlot() function. Heatmaps were generated showing the ratio of B and plasma cells that are positive for each gene of interest using the pheatmap package.

**Statistical analyses**

Analyses of Mass cytometry (Cytometry by time of flight (CyTOF)) of B cell subsets: Statistical comparison of cluster expression between populations was performed using two models. Firstly, differential cell abundance analysis was employed using the diffcyt-DA-edgeR package, a computational generalised linear mixed model that compensates for the variability in sample size and accounts for patient-patient variability [8]. Secondly, paired longitudinal samples were analysed by extracting cluster abundance per sample and comparing proportional fold change per sample using the Wilcoxon rank sum test. Correlation between abundance of B cell phenotypes was calculated using Spearman’s rank correlation test.

Data analysis of serum antibody isotype levels: Serum concentrations were analysed by calculating the relative abundance of each antibody isotype as a proportion of the total serum antibody concentration and to evaluate the ratio of IgG subtype to total IgG. For baseline samples, statistical comparisons between cohorts (e.g., melanoma patients versus healthy controls) were performed using the Mann Whitney U test for unpaired data (based on non-parametric distribution of the data). Paired analysis of longitudinal serum samples was made using Wilcoxon rank sum test.

Data analysis of autoantibody levels by immuno-mass spectrometry: Raw data derived from immuno-mass spectrometry experiments revealed potential auto-reactive antibody hits against 1,685 human proteins/antigens out of 13,028 human proteins tested across 64 patient samples derived from two technical duplicate injections. Analysis was performed in RStudio using packages: dplyr, ggplot2, and RVenn. Filtering criteria was applied to the raw data as detailed in Figure 2.5 to exclude: Proteins representing non-specific binding (i.e., immunoglobulin heavy and light chains; keratins; complement proteins); Proteins identified in the negative control (i.e., positive binding to anti-NIP IgG1); and Proteins identified in only one injection replicate (all samples were tested in two replicates, where one replicate was negative a mean could not be calculated and therefore the protein was excluded). The output from the filtering process identified in 144 candidate putative protein targets of auto-reactivity. The mean value of 2 technical replicates was used for downstream analysis. The filtered data was extracted from RStudio, and downstream analyses were performed using GraphPad Prism. Fold change comparisons were performed in for paired and unpaired analyses using the Mann Whitney U test and Wilcoxon rank sum test.

Survival analyses: Survival analyses were performed using the timepoint of pre-treatment blood sample as “day zero’ and the endpoints of ‘overall survival’ in days. Endpoint was death due to melanoma. Kaplan Meier plots were generated using Prism Graphpad software. Statistical significance of Kaplan Meier plots was evaluated using the log-rank test using GraphPad Prism software. Statistical significance of Kaplan Meier plots was evaluated using the log-rank test using GraphPad Prism software to define cut-off points of ‘high’ versus ‘low’ abundance of each cluster, Receiver Operator Curves (ROC) were generated (R package cutpointR was utilised to define optimal cut-off points). The log rank test was used for statistical analysis. The statistical package cutpointR was utilised to determine the optimal cut point for stratification of high versus low cluster abundance.

Univariate and pairwise-adjusted logistic regression analysis: To explore whether immune features associated with toxicity were independent of clinical covariates, we performed logistic regression models using toxicity status as the outcome. We conducted univariate and pairwise-adjusted models - adjusting each immune feature individually for age, sex, or tumour stage - to minimise overfitting given the small sample size [9]. Odds ratios, 95% confidence intervals, and P-values were extracted from each model and are reported in Supplementary Table 6 and Supplementary Table 7.

**Supplementary References**

1. Crescioli, S., et al., *B cell profiles, antibody repertoire and reactivity reveal dysregulated responses with autoimmune features in melanoma.* Nat Commun, 2023. **14**(1): p. 3378.

2. Harris, R.J., et al., *Enriched circulating and tumor-resident TGF-beta(+) regulatory B cells in patients with melanoma promote FOXP3(+) Tregs.* Oncoimmunology, 2022. **11**(1): p. 2104426.

3. Chauhan, J., et al., *Anti-cancer pro-inflammatory effects of an IgE antibody targeting the melanoma-associated antigen chondroitin sulfate proteoglycan 4.* Nat Commun, 2023. **14**(1): p. 2192.

4. Patel, A.J., et al., *Regulatory B cell repertoire defects predispose lung cancer patients to immune-related toxicity following checkpoint blockade.* Nat Commun, 2022. **13**(1): p. 3148.

5. Van Gassen, S., et al., *FlowSOM: Using self-organizing maps for visualization and interpretation of cytometry data.* Cytometry A, 2015. **87**(7): p. 636-45.

6. Nowicka, M., et al., *CyTOF workflow: differential discovery in high-throughput high-dimensional cytometry datasets.* F1000Res, 2017. **6**: p. 748.

7. Sade-Feldman, M., et al., *Defining T Cell States Associated with Response to Checkpoint Immunotherapy in Melanoma.* Cell, 2018. **175**(4): p. 998-1013 e20.

8. Weber, L.M., et al., *diffcyt: Differential discovery in high-dimensional cytometry via high-resolution clustering.* Commun Biol, 2019. **2**: p. 183.

9. Peduzzi, P., et al., *A simulation study of the number of events per variable in logistic regression analysis.* J Clin Epidemiol, 1996. **49**(12): p. 1373-9.
